# Supplementary material for: Can Wastewater Surveillance Enhance Genomic Tracking of Climate-Driven Pathogens?
Source: Microorganisms. 2025 Jan 28;13(2):294. doi: 10.3390/microorganisms13020294 (PMC11858121; doi:10.3390/microorganisms13020294)
Supplement: Supplementary file 1 [file microorganisms-13-00294-s001.zip › microorganisms-3306383-supplementary.pdf]

Table S1: Summary of pathogen detection urine, faeces and wastewater using various molecular methods. The table provides an overview of all pathogens from **Error! Reference source not found.** that were detected in published literature in urine, faeces and/or wastwater samples along with their employed methodologies. These methods include prior culturing or isolation followed by a molecular method such as PCR methods or next generation sequencing (NGS) and whole genome sequencing (WGS), the use of PCR methods, pathogen enrichment methods, including (nested) PCR and amplicon sequencing, in combination with sequencing, and finally metagenomics. The last column includes the method and sample that is used as gold standard for the detection of the human infection. The detection in urine and/or faeces was included because while some of these pathogens has not (yet) been found in wastewater, their detectability in urine or faeces indicates their potential to be found in wastewater. The font of waterborne diseases is blue, foodborne diseases green and vector–borne diseases in purple. Moreover, the cells of the pathogens are coloured according to the potential influence of climate change on the pathogens (low = green; medium = orange; high = red). (CCHF = Crimean–Congo haemorrhagic fever; STEC = Shiga toxin–producing E. coli, TBE = Tick–borne encephalitis; MAT = Microscopic Agglutination Test; Enzyme–Linked Immunosorbent Assay = ELISA; IFA = Indirect Immunofluorescence Antibody). If no published literature was available that confirms the possible detection of particular pathogens in urine, faeces or wastewater, this is indicated by "–".

Targets	Confirmed presence in ...			Detection of pathogens in wastewater with following methods				Gold standard detection in humans
	Urine	Faeces	Wastewater	Prior culturing or isolation + molecular method	PCR method	Pathogen enrichment methods + Sequencing	Metagenomics	
Cryptosporidium	–	[1–16]	[17–34]	–	PCR [17]<br>qPCR [18–22]<br>ddPCR [22, 23]	Nested PCR + Sanger sequencing [17, 24–29]<br>18S rRNA + Sanger sequencing [30]<br>18S rRNA + NGS [22, 31, 32]	Metagenomics [33, 34]	Microscopy on faeces [35]
Giardia	–	[2–4, 10, 11, 13–15, 36–43]	[17–19, 21, 24–29, 33, 34, 44–49]	–	PCR [17, 44]<br>qPCR [18, 19, 21, 45, 46]	Nested PCR + Sanger sequencing [17, 24–29, 45, 47, 48]<br>18S + NGS [49]	Metagenomics [33, 34]	Microscopy on faeces [50]
Leptospira	[51–61]		[62]	Culture + PCR [63]		PCR + Sanger [62]		MAT on serum [64]
**Vibrio**	–	[15, 65–86]	[33, 46, 81, 87–97]	Culture + PCR [87–89]<br>Culture + qPCR [90, 91]<br>Culture + 16S rDNA [90]	qPCR [46]	Sanger sequencing [90]<br>16srRNA + NGS [92–95]	Metagenomics [33, 96, 97]	Culture of faeces [98]
Clostridium botulinum	[99]	[100–109]	[110]	Culture + PCR [110]	–	–	–	Mouse bioassay of serum [111]
Toxoplasma	[112, 113]		[21, 32, 34, 114]	–	qPCR [21, 114]	18S rRNA + NGS [32]	Metagenomics [34]	Dye test of serum [115]
Listeria	[116]	[83, 84, 117–123]	[96]	–	–	–	Metagenomics [96]	Culture of blood, spinal fluid or placenta [124]

Campylobacter	–	[4, 13–15, 40–42, 84, 125–159]	[33, 96, 160–166]	Culture + PCR [160, 161, 167, 168]<br>PCR [162]<br>Culture + qPCR [163]<br>Culture 16s rDNA + Sanger sequencing [163]<br>Culture 16S rRNA + sequencing [164]	qPCR [164, 165]	16srRNA + NGS [166]	Metagenomics [33, 96]	Culture of faeces [169]
Hepatitis A	[170]	[170–181]	[1, 180, 182–198]	Isolation + WGS (Sanger) [182]	RT–PCR [182–187]<br>RT–qPCR [180, 183, 186, 188–195]	RT–PCR + Sanger [196]<br>Nested RT–PCR + Sanger sequencing [190, 191, 197]<br>Nested RT–PCR (SISP3) + NGS [193]<br>Random PCR amplification + NGS [199]	Metagenomics [198]	Antibody test on serum [200]
Legionella	[201–206]	–	[92, 95, 96, 166, 207, 208]	Culture 16S rDNA + sequencing [207]	qPCR [207, 208]	16srRNA + NGS [92, 95, 166]	Metagenomics [96]	Culture of lower respiratory secretions [209]
Salmonella	[210–217, 217]	[4, 13, 15, 16, 42, 43, 83, 85, 128, 130, 132, 134, 141, 142, 145–147, 149, 152, 155–159, 215, 218–229]	[33, 46, 89, 96, 97, 167, 230–239]	Culture + PCR [89, 167, 230]<br>Culture + WGS [231–233]	qPCR [46, 236–239]	16srRNA + NGS [95, 234]	Metagenomics [33, 96, 97, 235]	Culture of faeces or blood [240]
Shigella	[211, 241–243]	[8, 13, 42, 43, 83, 84, 128, 130–132, 134, 141, 142, 145–147, 149, 156, 159, 218, 219, 221,	[33, 33, 33, 46, 89, 97, 166, 235, 250–253]	Culture + PCR [89, 250–252]<br>Culture + qPCR [253]	qPCR [46]	16srRNA + NGS [166]	Metagenomics [33, 33, 33, 97, 235]	Culture of faeces [254]

		228, 244–249]						
STEC	[255]	[15, 155, 156, 159, 255–271]	[272]	Culture + PCR [272]	–	–	–	Culture of faeces [273]
Yersinia	[158, 217]	[130, 140, 152, 158, 159, 229, 229, 274–278]	[33, 162, 166, 234, 279]	–	PCR [162]	16s rRNA + NGS [166, 234, 279]	Metagenomics [33]	Culture of faeces [280]
Plasmodium (Malaria)	[281–287]	[288–290]	–	–	–	–	–	Microscopy on blood [291]
CCHF	[292–294]	–	–	–	–	–	–	ELISA and RT–PCR on blood [295]
Chikungunya virus	[296–302]	–	[303, 304]	–	RT–qPCR [303]	–	RNA–Seq [304]	RT–PCR of serum or plasma [305]
Dengue virus	[60, 302, 306–319]	–	[303, 320]	–	RT–qPCR [303] RT–ddPCR [320]	–	–	Molecular or serologic tests on serum [321]
Rift Valley fever	[322, 323]	[324]	–	–	–	–	–	RT–PCR or ELISA on blood [325]
TBE	[326–328]	–	–	–	–	–	–	ELISA on cerebrospinal fluid and serum [329]
West Nile fever	[302, 330, 330–341]	–	–	–	RT–qPCR [342]	–	–	Immunoassay on serum or cerebrospinal fluid [343]
Yellow Fever	[302, 322, 344, 344–347]	–	–	–	–	–	–	Serological tests on blood [348]
Zika virus	[307, 349–355]	–	[356]	–	qPCR [356]	–	–	Molecular or serologic tests on serum and urine [357]
**Borrelia (Lyme disease)**	[358–368]	–	–	–	–	–	–	Serological tests on blood [369]
**Leishmania (Visceral Leishmaniasis)**	[370–374]	–	–	–	–	–	–	Microscopy on bone marrow [375]
Bacillus anthracis (Anthrax)	–	–	–	–	–	–	–	Culture of clinical specimens [376]

Neisseria meningitidis (Meningococcal infection)	[377]	–	–	–	–	–	–	Culture on blood or cerebrospinal fluid [378]
Coxiella Burnetii (Q fever)	[379]	[379, 380]	[381]	–	qPCR [381]	–	–	IFA on serum [382]
Clostridium tetani (Tetanus)	–	[383]	–	–	–	–	–	Clinical symptoms [384]
Francisella tularensis (Tularaemia)	–	–	–	–	–	–	–	Culture of ulcer swabs or scrapings, lymph node aspirates or biopsies, pharyngeal swabs or respiratory specimen [385]
Hantavirus	[386–389]	–	–	–	–	–	–	ELISA on serum [390]

References

1. **Ng JSY, Ryan U, Trengove RD, Maker GL**. Development of an untargeted metabolomics method for the analysis of human faecal samples using Cryptosporidium-infected samples. *Molecular and Biochemical Parasitology* 2012;185:145–150.

2. **Manser M, Granlund M, Edwards H, Saez A, Petersen E, *et al.*** Detection of Cryptosporidium and Giardia in clinical laboratories in Europe—a comparative study. *Clinical Microbiology and Infection* 2014;20:O65–O71.

3. **ALEXANDER CL, CURRIE S, POLLOCK K, SMITH-PALMER A, JONES BL**. An audit of Cryptosporidium and Giardia detection in Scottish National Health Service Diagnostic Microbiology Laboratories. *Epidemiol Infect* 2017;145:1584–1590.

4. **Jex AR, Stanley KK, Lo W, Littman R, Verweij JJ, *et al.*** Detection of diarrhoeal pathogens in human faeces using an automated, robotic platform. *Mol Cell Probes* 2012;26:11–15.

5. **Bacchetti R, Connelly L, Browning L, Alexander CL**. Changing Molecular Profiles of Human Cryptosporidiosis Cases in Scotland as a Result of the Coronavirus Disease, COVID-19 Pandemic. *Br J Biomed Sci* 2023;80:11462.

6. **Dehkordy AB, Rafiei A, Alavi S, Latifi S**. Prevalence of cryptosporidium infection in immunocompromised patients, in South-west of iran, 2009-10. *Iran J Parasitol* 2010;5:42–47.

7. **Amatya R, Poudyal N, Gurung R, Khanal B**. Prevalence of cryptosporidium species in paediatric patients in Eastern Nepal. *Trop Doct* 2011;41:36–37.

8. **Kabayiza J-C, Andersson ME, Nilsson S, Baribwira C, Muhirwa G, *et al.*** Diarrhoeagenic microbes by real-time PCR in Rwandan children under 5 years of age with acute gastroenteritis. *Clin Microbiol Infect* 2014;20:O1128-1135.

9. **Ondriska F, Vrabcová I, Brinďáková S, Kváč M, Ditrich O, *et al.*** The first reported cases of human cryptosporidiosis caused by Cryptosporidium hominis in Slovak Republic. *Folia Microbiol (Praha)* 2013;58:69–73.

10. **Incani RN, Ferrer E, Hoek D, Ramak R, Roelfsema J, *et al.*** Diagnosis of intestinal parasites in a rural community of Venezuela: Advantages and disadvantages of using microscopy or RT-PCR. *Acta Trop* 2017;167:64–70.

11. **Polat E, Özdemir S, Sirekbasan S**. The Distribution of Intestinal Parasites in Patients Presenting to a University Hospital in Istanbul: A Seven-year Retrospective Analysis. *Turkiye Parazitol Derg* 2020;44:139–142.

12. **Rimšelienė G, Vold L, Robertson L, Nelke C, Søli K, *et al.*** An outbreak of gastroenteritis among schoolchildren staying in a wildlife reserve: thorough investigation reveals Norway's largest cryptosporidiosis outbreak. *Scand J Public Health* 2011;39:287–295.

13. **Giffen SR, Sadler JM, Miller MB**. QIAstat-Dx gastrointestinal panel and Luminex xTAG gastrointestinal pathogen panel comparative evaluation. *J Clin Microbiol* 2023;61:e0085923.

14. **Marcenac P, Traoré A, Kim S, Prentice-Mott G, Berendes DM, *et al.*** Giardia Detection and Codetection With Other Enteric Pathogens in Young Children in the Vaccine Impact on Diarrhea in Africa (VIDA) Case-Control Study: 2015-2018. *Clin Infect Dis* 2023;76:S106–S113.

15. **Chau ML, Hartantyo SHP, Yap M, Kang JSL, Aung KT, *et al.*** Diarrheagenic pathogens in adults attending a hospital in Singapore. *BMC Infect Dis* 2016;16:32.

16. **Bejide OS, Odebode MA, Ogunbosi BO, Adekanmbi O, Akande KO, *et al.*** Diarrhoeal pathogens in the stools of children living with HIV in Ibadan, Nigeria. *Front Cell Infect Microbiol* 2023;13:1108923.

17. **Fan Y, Wang X, Yang R, Zhao W, Li N, *et al.*** Molecular characterization of the waterborne pathogens Cryptosporidium spp., Giardia duodenalis, Enterocytozoon bieneusi, Cyclospora cayetanensis and Eimeria spp. in wastewater and sewage in Guangzhou, China. *Parasit Vectors* 2021;14:66.

18. **Dungan RS, Klein M, Leytem AB**. Quantification of Bacterial Indicators and Zoonotic Pathogens in Dairy Wastewater Ponds. *Appl Environ Microbiol* 2012;78:8089–8095.

19. **Ladeia WA, Martins FDC, Nino B de SL, Silvério A da C, da Silva AC, *et al.*** High occurrence of viable forms of Cryptosporidium and Giardia in domestic sewage from an agricultural region of Brazil. *J Water Health* 2022;20:1405–1415.

20. **Li N, Neumann NF, Ruecker N, Alderisio KA, Sturbaum GD, *et al.*** Development and Evaluation of Three Real-Time PCR Assays for Genotyping and Source Tracking Cryptosporidium spp. in Water. *Appl Environ Microbiol* 2015;81:5845–5854.

21. **Marangi M, Giangaspero A, Lacasella V, Lonigro A, Gasser RB**. Multiplex PCR for the detection and quantification of zoonotic taxa of Giardia, Cryptosporidium and Toxoplasma in wastewater and mussels. *Mol Cell Probes* 2015;29:122–125.

22. **Zahedi A, Gofton AW, Greay T, Monis P, Oskam C, *et al.*** Profiling the diversity of Cryptosporidium species and genotypes in wastewater treatment plants in Australia using next generation sequencing. *Science of The Total Environment* 2018;644:635–648.

23. **Mthethwa NP, Amoah ID, Reddy P, Bux F, Kumari S**. Development and evaluation of a molecular based protocol for detection and quantification of Cryptosporidium spp. in wastewater. *Experimental Parasitology* 2022;234:108216.

24. **Ben Ayed L, Yang W, Widmer G, Cama V, Ortega Y, *et al.*** Survey and genetic characterization of wastewater in Tunisia for Cryptosporidium spp., Giardia

duodenalis, Enterocytozoon bieneusi, Cyclospora cayetanensis and Eimeria spp. *Journal of Water and Health* 2012;10:431–444.

25. **Huang C, Hu Y, Wang L, Wang Y, Li N, *et al.*** Environmental Transport of Emerging Human-Pathogenic Cryptosporidium Species and Subtypes through Combined Sewer Overflow and Wastewater. *Appl Environ Microbiol* 2017;83:e00682-17.

26. **Jiang W, Roellig DM, Li N, Wang L, Guo Y, *et al.*** Contribution of hospitals to the occurrence of enteric protists in urban wastewater. *Parasitol Res* 2020;119:3033–3040.

27. **Khouja LBA, Cama V, Xiao L**. Parasitic contamination in wastewater and sludge samples in Tunisia using three different detection techniques. *Parasitol Res* 2010;107:109–116.

28. **Kitajima M, Haramoto E, Iker BC, Gerba CP**. Occurrence of Cryptosporidium, Giardia, and Cyclospora in influent and effluent water at wastewater treatment plants in Arizona. *Science of The Total Environment* 2014;484:129–136.

29. **Ulloa-Stanojlović FM, Aguiar B, Jara LM, Sato MIZ, Guerrero JA, *et al.*** Occurrence of Giardia intestinalis and Cryptosporidium sp. in wastewater samples from São Paulo State, Brazil, and Lima, Peru. *Environ Sci Pollut Res* 2016;23:22197–22205.

30. **HATAM-NAHAVANDI K, MOHEBALI M, MAHVI A-H, KESHAVARZ H, NAJAFIAN H-R, *et al.*** Microscopic and Molecular Detection of Cryptosporidium andersoni and Cryptosporidium xiaoi in Wastewater Samples of Tehran Province, Iran. *Iran J Parasitol* 2016;11:499–506.

31. **Moreno Y, Moreno-Mesonero L, Amorós I, Pérez R, Morillo JA, *et al.*** Multiple identification of most important waterborne protozoa in surface water used for irrigation purposes by 18S rRNA amplicon-based metagenomics. *International Journal of Hygiene and Environmental Health* 2018;221:102–111.

32. **Maritz JM, Rogers KH, Rock TM, Liu N, Joseph S, *et al.*** An 18S rRNA Workflow for Characterizing Protists in Sewage, with a Focus on Zoonotic Trichomonads. *Microb Ecol* 2017;74:923–936.

33. **Hendriksen RS, Lukjancenko O, Munk P, Hjelmsø MH, Verani JR, *et al.*** Pathogen surveillance in the informal settlement, Kibera, Kenya, using a metagenomics approach. *PLoS One* 2019;14:e0222531.

34. **Ariyadasa S, Taylor W, Weaver L, McGill E, Billington C, *et al.*** Nonbacterial Microflora in Wastewater Treatment Plants: an Underappreciated Potential Source of Pathogens. *Microbiol Spectr*;11:e00481-23.

35. **Aboelsoued, D.; Abdel Megeed, K.N.** Diagnosis and Control of Cryptosporidiosis in Farm Animals. *J Parasit Dis* **2022**, *46*, 1133–1146, doi:10.1007/s12639-022-01513-2..

36. **Asher AJ, Waldron LS, Power ML**. Evaluation of a PCR protocol for sensitive detection of Giardia intestinalis in human faeces. *Parasitol Res* 2012;110:853–858.

37. **Lass A, Karanis P, Korzeniewski K**. First detection and genotyping of Giardia intestinalis in stool samples collected from children in Ghazni Province, eastern Afghanistan and evaluation of the PCR assay in formalin-fixed specimens. *Parasitol Res* 2017;116:2255–2264.

38. **Menu E, Mary C, Toga I, Raoult D, Ranque S, *et al.*** A hospital qPCR-based survey of 10 gastrointestinal parasites in routine diagnostic screening, Marseille, France. *Epidemiol Infect* 2019;147:e100.

39. **Sarkari B, Ashrafmansori A, Hatam GR, Motazedian MH, Asgari Q, *et al.*** Genotyping of Giardia lamblia isolates from human in southern Iran. *Trop Biomed* 2012;29:366–371.

40. **Barati M, Taghipour A, Bakhshi B, Shams S, Pirestani M**. Prevalence of intestinal parasitic infections and Campylobacter spp. among children with gastrointestinal disorders in Tehran, Iran. *Parasite Epidemiol Control* 2021;13:e00207.

41. **García-Sánchez C, García-Rodríguez J, Ruiz-Carrascoso G**. Clinical and microbiological findings of recurrent Campylobacter spp. gastroenteritis in a tertiary care hospital. *Enferm Infecc Microbiol Clin (Engl Ed)* 2023;S2529-993X(23)00035–7.

42. **Ndjangangoye NK, Lekana-Douki SE, Oyegue-Liabagui SL, Kouna LC, Ndong Ndong KA, *et al.*** Molecular Prevalence of Diarrheal Pathogens in Children with Acute Diarrhea in Southeastern Gabon. *Am J Trop Med Hyg* 2023;108:829–836.

43. **Abbasi E, van Belkum A, Ghaznavi-Rad E**. Common Etiological Agents in Adult Patients with Gastroenteritis from Central Iran. *Microb Drug Resist* 2022;28:1043–1055.

44. **Fernandes LN, de Souza PP, de Araújo RS, Razzolini MTP, Soares RM, *et al.*** Detection of assemblages A and B of Giardia duodenalis in water and sewage from São Paulo state, Brazil. *Journal of Water and Health* 2011;9:361–367.

45. **Moreno-Mesonero L, Amorós I, Moreno Y, Alonso JL**. Simultaneous detection of less frequent waterborne parasitic protozoa in reused wastewater using amplicon sequencing and qPCR techniques. *Journal of Environmental Management* 2022;314:115029.

46. **Liu P, Amin N, Miah R, Foster T, Raj S, *et al.*** A method for correcting underestimation of enteric pathogen genome quantities in environmental samples. *Journal of Microbiological Methods* 2021;189:106320.

47. **Chowdhari S, Rana S, Rana S, Morrison CM, Abney SE, *et al.*** Quantitative Assessment of Microbial Pathogens and Indicators of Wastewater Treatment

Performance for Safe and Sustainable Water Reuse in India. *Microbiol Spectr*;10:e01720-22.

48. **Hatam-Nahavandi K, Mohebali M, Mahvi A-H, Keshavarz H, Mirjalali H, *et al.*** Subtype analysis of Giardia duodenalis isolates from municipal and domestic raw wastewaters in Iran. *Environ Sci Pollut Res* 2017;24:12740–12747.

49. **Rozo-Montoya N, Bedoya-Urrego K, Alzate JF**. Monitoring potentially pathogenic protists in sewage sludge using Metataxonomics. *Food Waterborne Parasitol* 2023;33:e00210.

50. **Pouryousef, A.; Fararouei, M.; Sarkari, B.** Antigen-Based Diagnosis of Human Giardiasis: A Systematic Review and Meta-Analysis. *Iran J Parasitol* **2023**, *18*, 140–154, doi:10.18502/ijpa.v18i2.13180.

51. **Ahmed AA, Goris MGA, Meijer MC**. Development of lipL32 real-time PCR combined with an internal and extraction control for pathogenic Leptospira detection. *PLoS One* 2020;15:e0241584.

52. **Allan KJ, Maze MJ, Galloway RL, Rubach MP, Biggs HM, *et al.*** Molecular Detection and Typing of Pathogenic Leptospira in Febrile Patients and Phylogenetic Comparison with Leptospira Detected among Animals in Tanzania. *Am J Trop Med Hyg* 2020;103:1427–1434.

53. **Esteves LM, Bulhões SM, Branco CC, Carreira T, Vieira ML, *et al.*** Diagnosis of Human Leptospirosis in a Clinical Setting: Real-Time PCR High Resolution Melting Analysis for Detection of Leptospira at the Onset of Disease. *Sci Rep* 2018;8:9213.

54. **Iwasaki H, Chagan-Yasutan H, Leano PSA, Koizumi N, Nakajima C, *et al.*** Combined antibody and DNA detection for early diagnosis of leptospirosis after a disaster. *Diagn Microbiol Infect Dis* 2016;84:287–291.

55. **Othman S, Lee P-Y, Lam J-Y, Philip N, Azhari NN, *et al.*** A versatile isothermal amplification assay for the detection of leptospires from various sample types. *PeerJ* 2022;10:e12850.

56. **Shukla S, Mittal V, Karoli R, Singh P, Singh A**. Leptospirosis in central & eastern Uttar Pradesh, an underreported disease: A prospective cross-sectional study. *Indian J Med Res* 2022;155:66–72.

57. **Villumsen S, Pedersen R, Borre MB, Ahrens P, Jensen JS, *et al.*** Novel TaqMan® PCR for detection of Leptospira species in urine and blood: pit-falls of in silico validation. *J Microbiol Methods* 2012;91:184–190.

58. **Weiss S, Menezes A, Woods K, Chanthongthip A, Dittrich S, *et al.*** An Extended Multilocus Sequence Typing (MLST) Scheme for Rapid Direct Typing of Leptospira from Clinical Samples. *PLoS Negl Trop Dis* 2016;10:e0004996.

59. **Le-Viet N, Le V-N, Chung H, Phan D-T, Phan Q-D, *et al.*** Prospective case-control analysis of the aetiologies of acute undifferentiated fever in Vietnam. *Emerg Microbes Infect* 2019;8:339–352.

60. **Ashaiba A, Arun AB, Prasad KS, Tellis RC**. A clinical pilot study for the detection of sphingomyelinase in leptospirosis patient’s urine at tertiary care hospital. *Heliyon* 2023;9:e21138.

61. **Søndergaard MM, Tursunovic A, Thye-Rønn P, Bang JC, Hansen IMJ**. Leptospirosis-Associated Severe Pulmonary Hemorrhagic Syndrome with Lower Back Pain as an Initial Symptom. *Am J Case Rep* 2016;17:883–886.

62. **Agboola TD, Nmema EE, Odetoyin BW**. Distribution and antibiogram of Vibrio species from hospital wastewater in Southwest, Nigeria. *Pan Afr Med J* 2023;45:80.

63. **Igbinosa EO, Obi CL, Okoh AI**. Seasonal abundance and distribution of Vibrio species in the treated effluent of wastewater treatment facilities in suburban and urban communities of Eastern Cape Province, South Africa. *J Microbiol* 2011;49:224–232.

64. **Pinto, G.V.; Senthilkumar, K.; Rai, P.; Kabekkodu, S.P.; Karunasagar, I.; Kumar, B.K.** Current Methods for the Diagnosis of Leptospirosis: Issues and Challenges. *Journal of Microbiological Methods* **2022**, *195*, 106438, doi:10.1016/j.mimet.2022.106438..

65. **Afum T, Asandem DA, Asare P, Asante-Poku A, Mensah GI, *et al.*** Diarrhea-Causing Bacteria and Their Antibiotic Resistance Patterns Among Diarrhea Patients From Ghana. *Front Microbiol* 2022;13:894319.

66. **Alexandrova L, Haque F, Rodriguez P, Marrazzo AC, Grembi JA, *et al.*** Identification of Widespread Antibiotic Exposure in Patients With Cholera Correlates With Clinically Relevant Microbiota Changes. *J Infect Dis* 2019;220:1655–1666.

67. **Al-Sa’ady AT, Baqer KA, Al-Salim ZKS**. Molecular detection and phylogenetic analysis of Vibrio cholerae genotypes in Hillah, Iraq. *New Microbes New Infect* 2020;37:100739.

68. **Bodhidatta L, Anuras S, Sornsakrin S, Suksawad U, Serichantalergs O, *et al.*** Epidemiology and etiology of Traveler’s diarrhea in Bangkok, Thailand, a case-control study. *Trop Dis Travel Med Vaccines* 2019;5:9.

69. **Chen X, Zhu Q, Liu Y, Wang R, Xie H, *et al.*** Pathogenic Characteristics of and Variation in Vibrio parahaemolyticus Isolated from Acute Diarrhoeal Patients in Southeastern China from 2013 to 2017. *Infect Drug Resist* 2020;13:1307–1318.

70. **Chowdhury G, Senapati T, Das B, Kamath A, Pal D, *et al.*** Laboratory evaluation of the rapid diagnostic tests for the detection of Vibrio cholerae O1 using diarrheal samples. *PLoS Negl Trop Dis* 2021;15:e0009521.

71. **Fattel L, Panossian B, Salloum T, Abboud E, Tokajian S**. Genomic Features of Vibrio parahaemolyticus from Lebanon and Comparison to Globally Diverse Strains by Whole-Genome Sequencing. *Foodborne Pathog Dis* 2019;16:778–787.

72. **Gao Q, Liu H, Yu W, Wang Z, Yang Y, *et al.*** Pathogenetic characteristics of infectious diarrhea in Yantai City, Shandong Province, 2018-2019. *Front Public Health* 2023;11:1195118.

73. **Jin D, Luo Y, Zheng M, Li H, Zhang J, *et al.*** Quantitative detection of Vibrio cholera toxin by real-time and dynamic cytotoxicity monitoring. *J Clin Microbiol* 2013;51:3968–3974.

74. **Li Z, Guan H, Wang W, Gao H, Feng W, *et al.*** Development of a Rapid and Fully Automated Multiplex Real-Time PCR Assay for Identification and Differentiation of Vibrio cholerae and Vibrio parahaemolyticus on the BD MAX Platform. *Front Cell Infect Microbiol* 2021;11:639473.

75. **Owusu M, Nkrumah B, Acheampong G, Mensah EK, Komei AA-K, *et al.*** Improved detection of microbiological pathogens: role of partner and non-governmental organizations. *BMC Infect Dis* 2021;21:303.

76. **Sah SK, Basnet S, Shrestha S, Ghale K, Tamang S, *et al.*** Burden of Shigella spp and Vibrio spp, and their antibiotic sensitivity pattern in the patients with acute gastroenteritis in tertiary care hospital in Nepal. *BMC Res Notes* 2019;12:699.

77. **Sarker MHR, Moriyama M, Rahman MM, Das SK, Uzzaman MN, *et al.*** Characteristics of Rotavirus, ETEC, and Vibrio Cholerae Among Under 2-year Children Attending an Urban Diarrheal Disease Hospital in Bangladesh. *J Prim Care Community Health* 2021;12:21501327211049120.

78. **Simner PJ, Oethinger M, Stellrecht KA, Pillai DR, Yogev R, *et al.*** Multisite Evaluation of the BD Max Extended Enteric Bacterial Panel for Detection of Yersinia enterocolitica, Enterotoxigenic Escherichia coli, Vibrio, and Plesiomonas shigelloides from Stool Specimens. *J Clin Microbiol* 2017;55:3258–3266.

79. **Thapa Shrestha U, Adhikari N, Maharjan R, Banjara MR, Rijal KR, *et al.*** Multidrug resistant Vibrio cholerae O1 from clinical and environmental samples in Kathmandu city. *BMC Infect Dis* 2015;15:104.

80. **Zamani P, Sajedi RH, Hosseinkhani S, Zeinoddini M**. Hybridoma as a specific, sensitive, and ready to use sensing element: a rapid fluorescence assay for detection of Vibrio cholerae O1. *Anal Bioanal Chem* 2016;408:6443–6451.

81. **Zereen F, Akter S, Sobur MA, Hossain MT, Rahman MT**. Molecular detection of Vibrio cholerae from human stool collected from SK Hospital, Mymensingh, and their antibiogram. *J Adv Vet Anim Res* 2019;6:451–455.

82. **Zhang P, Wu X, Yuan R, Yan W, Xu D, *et al.*** Emergence and predominance of a new serotype of Vibrio parahaemolyticus in Huzhou, China. *Int J Infect Dis* 2022;122:93–98.

83. **Rundell MS, Pingle M, Das S, Hussain A, Ocheretina O, *et al.*** A multiplex PCR/LDR assay for simultaneous detection and identification of the NIAID category B bacterial food and water-borne pathogens. *Diagn Microbiol Infect Dis* 2014;79:135–140.

84. **Hu Q, Lyu D, Shi X, Jiang Y, Lin Y, *et al.*** A modified molecular beacons-based multiplex real-time PCR assay for simultaneous detection of eight foodborne pathogens in a single reaction and its application. *Foodborne Pathog Dis* 2014;11:207–214.

85. **Takahashi E, Motooka D, Nakamura S, Miyoshi S-I, Chowdhury G, *et al.*** Metagenomic analysis of diarrheal stools in Kolkata, India, indicates the possibility of subclinical infection of Vibrio cholerae O1. *Sci Rep* 2022;12:19473.

86. **Debes AK, Ateudjieu J, Guenou E, Ebile W, Sonkoua IT, *et al.*** Clinical and Environmental Surveillance for Vibrio cholerae in Resource Constrained Areas: Application During a 1-Year Surveillance in the Far North Region of Cameroon. *The American Journal of Tropical Medicine and Hygiene* 2016;94:537–543.

87. **Mavhungu M, Digban TO, Nwodo UU**. Incidence and Virulence Factor Profiling of Vibrio Species: A Study on Hospital and Community Wastewater Effluents. *Microorganisms* 2023;11:2449.

88. **Okoh AI, Sibanda T, Nongogo V, Adefisoye M, Olayemi OO, *et al.*** Prevalence and characterisation of non-cholerae Vibrio spp. in final effluents of wastewater treatment facilities in two districts of the Eastern Cape Province of South Africa: implications for public health. *Environ Sci Pollut Res Int* 2015;22:2008–2017.

89. **Teklehaimanot GZ, Coetzee MAA, Momba MNB**. Faecal pollution loads in the wastewater effluents and receiving water bodies: a potential threat to the health of Sedibeng and Soshanguve communities, South Africa. *Environ Sci Pollut Res* 2014;21:9589–9603.

90. **Cañigral I, Moreno Y, Alonso JL, González A, Ferrús MA**. Detection of Vibrio vulnificus in seafood, seawater and wastewater samples from a Mediterranean coastal area. *Microbiological Research* 2010;165:657–664.

91. **Aguiar-Oliveira M de L, Campos A, R. Matos A, Rigotto C, Sotero-Martins A, *et al.*** Wastewater-Based Epidemiology (WBE) and Viral Detection in Polluted Surface Water: A Valuable Tool for COVID-19 Surveillance—A Brief Review. *IJERPH* 2020;17:9251.

92. **Huang K, Mao Y, Zhao F, Zhang X-X, Ju F, *et al.*** Free-living bacteria and potential bacterial pathogens in sewage treatment plants. *Appl Microbiol Biotechnol* 2018;102:2455–2464.

93. **Baraka V, Andersson T, Makenga G, Francis F, Minja DTR, *et al.*** Unveiling Rare Pathogens and Antibiotic Resistance in Tanzanian Cholera Outbreak Waters. *Microorganisms* 2023;11:2490.

94. **Selvarajan R, Sibanda T, Pandian J, Mearns K**. Taxonomic and Functional Distribution of Bacterial Communities in Domestic and Hospital Wastewater System: Implications for Public and Environmental Health. *Antibiotics (Basel)* 2021;10:1059.

95. **Cui Q, Huang Y, Wang H, Fang T**. Diversity and abundance of bacterial pathogens in urban rivers impacted by domestic sewage. *Environmental Pollution* 2019;249:24–35.

96. **Tang Y, Liang Z, Li G, Zhao H, An T**. Metagenomic profiles and health risks of pathogens and antibiotic resistance genes in various industrial wastewaters and the associated receiving surface water. *Chemosphere* 2021;283:131224.

97. **Ekwanzala MD, Dewar JB, Momba MNB**. Environmental resistome risks of wastewaters and aquatic environments deciphered by shotgun metagenomic assembly. *Ecotoxicology and Environmental Safety* 2020;197:110612.

98. **Chowdhury, F.; Ross, A.G.; Islam, M.T.; McMillan, N.A.J.; Qadri, F.** Diagnosis, Management, and Future Control of Cholera. *Clinical Microbiology Reviews* **2022**, *35*, e00211-21, doi:10.1128/cmr.00211-21.

99. **Wang J, Xu H, Zhang C, Chen J, Wang C, *et al.*** Serotype Features of 17 Suspected Cases of Foodborne Botulism in China 2019–2022 Revealed by a Multiplex Immuno-Endopep-MS Method. *Front Microbiol* 2022;13:869874.

100. **Aureli P, Di Cunto M, Maffei A, De Chiara G, Franciosa G, *et al.*** An outbreak in Italy of botulism associated with a dessert made with mascarpone cream cheese. *Eur J Epidemiol* 2000;16:913–918.

101. **Grant KA, Nwarfor I, Mpamugo O, Mithani V, Lister P, *et al.*** Report of two unlinked cases of infant botulism in the UK in October 2007. *J Med Microbiol* 2009;58:1601–1606.

102. **Brett MM, McLauchlin J, Harris A, O'Brien S, Black N, *et al.*** A case of infant botulism with a possible link to infant formula milk powder: evidence for the presence of more than one strain of Clostridium botulinum in clinical specimens and food. *J Med Microbiol* 2005;54:769–776.

103. **Raphael BH, Joseph LA, McCroskey LM, Lúquez C, Maslanka SE**. Detection and differentiation of Clostridium botulinum type A strains using a focused DNA microarray. *Mol Cell Probes* 2010;24:146–153.

104. **Hill BJ, Skerry JC, Smith TJ, Arnon SS, Douek DC**. Universal and specific quantitative detection of botulinum neurotoxin genes. *BMC Microbiol* 2010;10:267.

105. **Lin YJ, Li F, Su J, Meng WW, Tian T, *et al.*** [Tracing investigation and analysis of a Clostridium botulinum food poisoning incident in Bayingolin Mongolian Autonomous Prefecture, Xinjiang]. *Zhonghua Yu Fang Yi Xue Za Zhi* 2022;56:541–544.

106. **Mazuet C, Yoon E-J, Boyer S, Pignier S, Blanc T, *et al.*** A penicillin- and metronidazole-resistant Clostridium botulinum strain responsible for an infant botulism case. *Clin Microbiol Infect* 2016;22:644.e7-644.e12.

107. **Rosen HE, Kimura AC, Crandall J, Poe A, Nash J, *et al.*** Foodborne Botulism Outbreak Associated With Commercial Nacho Cheese Sauce From a Gas Station Market. *Clin Infect Dis* 2020;70:1695–1700.

108. **Rafie S, Salmanzadeh S, Mehramiri A, Nejati A**. Botulism Outbreak in a Family after Ingestion of Locally Produced Cheese. *Iran J Med Sci* 2017;42:201–204.

109. **Casado-Ruiz V, Cano A, Suárez-López A, Muriana D, Guanyabens-Buscà N, *et al.*** Food borne-botulism during SARS-CoV-2 pandemic time. A case and a possible familial outbreak in Barcelona. *Rev Neurol* 2022;75:71–74.

110. **Anza I, Vidal D, Laguna C, Díaz-Sánchez S, Sánchez S, *et al.*** Eutrophication and Bacterial Pathogens as Risk Factors for Avian Botulism Outbreaks in Wetlands Receiving Effluents from Urban Wastewater Treatment Plants. *Appl Environ Microbiol* 2014;80:4251–4259.

111. **Center for Disease Control and Prevention**. Clinical Guidelines for Diagnosis and Treatment of Botulism, 2021. *MMWR Recomm Rep*;70. Epub ahead of print 2021. DOI: 10.15585/mmwr.rr7002a1.

112. **Naik KV, Mishra A, Panda S, Sinha A, Padhi M, *et al.*** Seropositivity of Chlamydia trachomatis & Toxoplasma gondii among male partners of infertile couples in Odisha, India: A facility-based exploratory study. *Indian J Med Res* 2022;156:681–684.

113. **Zauli DAG, de Menezes CLP, de Oliveira CL**. Development and padronization of three multiplex PCRs for the diagnosis of Chlamydia trachomatis, Toxoplasma gondii, herpes simplex viruses 1 and 2, and Cytomegalovirus. *Mol Biotechnol* 2013;54:1004–1009.

114. **Lass A, Kontogeorgos I, Ma L, Zhang X, Li X, *et al.*** Investigation of Toxoplasma gondii in wastewater and surface water in the Qinghai-Tibet Plateau, China using real-time PCR and multilocus genotyping. *Sci Rep* 2022;12:5428.

115. **Kim, M.; Park, S.J.; Park, H.** Trend in Serological and Molecular Diagnostic Methods for Toxoplasma Gondii Infection. *Eur J Med Res* **2024**, *29*, 520, doi:10.1186/s40001-024-02055-4.

116. **Al-Mayahi FSA, Jaber SM**. Multiple drug resistance of Listeria monocytogenes isolated from aborted women by using serological and molecular techniques in Diwaniyah city/Iraq. *Iran J Microbiol* 2020;12:305–312.

117. **Hoyles L, Honda H, Logan NA, Halket G, La Ragione RM, *et al.*** Recognition of greater diversity of Bacillus species and related bacteria in human faeces. *Res Microbiol* 2012;163:3–13.

118. **Olier MW, Pierre F, Lemaı Tre J-P, Divies C, Rousset A, *et al.*** Assessment of the pathogenic potential of two Listeria monocytogenes human faecal carriage isolates. *Microbiology (Reading)* 2002;148:1855–1862.

119. **Cobb CA, Curtis GD, Bansi DS, Slade E, Mehal W, *et al.*** Increased prevalence of Listeria monocytogenes in the faeces of patients receiving long-term H2-antagonists. *Eur J Gastroenterol Hepatol* 1996;8:1071–1074.

120. **Jensen A**. Listeria monocytogenes isolation from human faecal specimens: experiments with the selective media, PALCAM and L-PALCAMY. *Lett Appl Microbiol* 1993;16:32–35.

121. **Hafner L, Pichon M, Burucoa C, Nusser SHA, Moura A, *et al.*** Listeria monocytogenes faecal carriage is common and depends on the gut microbiota. *Nat Commun* 2021;12:6826.

122. **Madajczak G, Szych J, Wójcik B, Mąka Ł, Formińska K**. Validation of direct plating of a stool sample as a method for Listeria monocytogenes detection. *Ann Agric Environ Med* 2012;19:69–74.

123. **Grif K, Patscheider G, Dierich MP, Allerberger F**. Incidence of fecal carriage of Listeria monocytogenes in three healthy volunteers: a one-year prospective stool survey. *Eur J Clin Microbiol Infect Dis* 2003;22:16–20.

124. **Ravindhiran, R.; Sivarajan, K.; Sekar, J.N.; Murugesan, R.; Dhandapani, K.** Listeria Monocytogenes an Emerging Pathogen: A Comprehensive Overview on Listeriosis, Virulence Determinants, Detection, and Anti-Listerial Interventions. *Microb Ecol* **2023**, *86*, 2231–2251, doi:10.1007/s00248-023-02269-9.

125. **Nafarrate I, Lasagabaster A, Sevillano E, Mateo E**. Prevalence, molecular typing and antimicrobial susceptibility of Campylobacter spp. isolates in northern Spain. *J Appl Microbiol* 2021;130:1368–1379.

126. **Ferreira S, Júlio C, Queiroz JA, Domingues FC, Oleastro M**. Molecular diagnosis of Arcobacter and Campylobacter in diarrhoeal samples among Portuguese patients. *Diagn Microbiol Infect Dis* 2014;78:220–225.

127. **Leblanc-Maridor M, Beaudeau F, Seegers H, Denis M, Belloc C**. Rapid identification and quantification of Campylobacter coli and Campylobacter jejuni by real-time PCR in pure cultures and in complex samples. *BMC Microbiol* 2011;11:113.

128. **Koziel M, Corcoran D, O'Callaghan I, Sleator RD, Lucey B**. Validation of the EntericBio Panel II® multiplex polymerase chain reaction system for detection of Campylobacter spp., Salmonella spp., Shigella spp., and verotoxigenic E. coli for use in a clinical diagnostic setting. *Diagn Microbiol Infect Dis* 2013;75:46–49.

129. **Quetz J da S, Lima IFN, Havt A, Prata MMG, Cavalcante PA, *et al.*** Campylobacter jejuni infection and virulence-associated genes in children with moderate to severe diarrhoea admitted to emergency rooms in northeastern Brazil. *J Med Microbiol* 2012;61:507–513.

130. **Truong J, Cointe A, Le Roux E, Bidet P, Michel M, *et al.*** Clinical impact of a gastrointestinal PCR panel in children with infectious diarrhoea. *Arch Dis Child* 2022;107:601–605.

131. **Montasser K, Osman HA, Abozaid H, Khalil HS, Hatem Amer W, *et al.*** Multiplex PCR: Aid to more-timely and directed therapeutic intervention for patients with infectious gastroenteritis. *Medicine (Baltimore)* 2022;101:e31022.

132. **Alzaher MZ, Almugahwi AA, Almulla AA, Almeer HH, Alshammasi MM, *et al.*** Diagnostic yield of stool culture and probable predictive factors. *Acta Biomed* 2022;93:e2022302.

133. **Ghorbani Marghmaleki E, Ahmadi A, Arjomandzadegan M, Akbari M, Karamghoshchi A**. Molecular Detection of Campylobacter Species: Comparision of 16SrRNA with slyD, cadF, rpoA, and dnaJ Sequencing. *Rep Biochem Mol Biol* 2020;9:257–263.

134. **Newman KL, Newman GS, Cybulski RJ, Fang FC**. Gastroenteritis in Men Who Have Sex With Men in Seattle, Washington, 2017-2018. *Clin Infect Dis* 2020;71:109–115.

135. **Teksoy N, Ilktac M, Ongen B**. Investigating the Significance of Non-jejuni/coli Campylobacter Strains in Patients with Diarrhea. *Healthcare (Basel)* 2023;11:2562.

136. **Zeinhom MMA, Abdel-Latef GK, Corke H**. Prevalence, Characterization, and Control of Campylobacter jejuni Isolated from Raw Milk, Cheese, and Human Stool Samples in Beni-Suef Governorate, Egypt. *Foodborne Pathog Dis* 2021;18:322–330.

137. **Ganji L, Shirazi MH, Ebrahimi-Daryani N, Eslami P, Rahbar M, *et al.*** Carriage of CdtB Encoding Campylobacter spp., Salmonella enterica, and Yersinia entercolitica in Patients with Gastroenteritis and Irritable Bowel Syndrome. *Dig Dis Sci* 2022;67:5522–5528.

138. **Hizlisoy H, Sagiroglu P, Barel M, Dishan A, Gungor C, *et al.*** Campylobacter jejuni and Campylobacter coli in human stool samples: antibiotic resistance profiles, putative virulence determinants and molecular characterization of the isolates. *World J Microbiol Biotechnol* 2023;39:353.

139. **Sayed ASM, Ibrahim AI, Sobhy MM, Elmahallawy EK, Alsowayeh N, *et al.*** Circulation of thermophilic Campylobacter in pigeons, turkeys, and humans at live bird markets in Egypt. *Front Vet Sci* 2023;10:1150077.

140. **Porte L, Pérez C, Barbé M, Varela C, Vollrath V, *et al.*** Campylobacter spp. Prevalence in Santiago, Chile: A Study Based on Molecular Detection in Clinical Stool Samples from 2014 to 2019. *Pathogens* 2023;12:504.

141. **Amanpour Z, Kouhsari E, Pakzad I, Kenarkoohi A, Sadeghifard N**. Simultaneous Molecular Detection of Common Bacterial Enteropathogens in Children with Diarrhea by Multiplex-PCR Assay. *Clin Lab*;67. Epub ahead of print 1 June 2021. DOI: 10.7754/Clin.Lab.2020.201046.

142. **Peterson C-L, Alexander D, Chen JC-Y, Adam H, Walker M, *et al.*** Clinical Metagenomics Is Increasingly Accurate and Affordable to Detect Enteric Bacterial Pathogens in Stool. *Microorganisms* 2022;10:441.

143. **Buss JE, Thacker E, Santiago M**. Culture Methods to Determine the Limit of Detection and Survival in Transport Media of Campylobacter Jejuni in Human Fecal Specimens. *J Vis Exp*. Epub ahead of print 10 March 2020. DOI: 10.3791/60457.

144. **Terefe Y, Deblais L, Ghanem M, Helmy YA, Mummed B, *et al.*** Co-occurrence of Campylobacter Species in Children From Eastern Ethiopia, and Their Association With Environmental Enteric Dysfunction, Diarrhea, and Host Microbiome. *Front Public Health* 2020;8:99.

145. **Hlashwayo DF, Noormahomed EV, Bahule L, Benson C, Schooley RT, *et al.*** Microbiological assessment reveals that Salmonella, Shigella and Campylobacter infections are widespread in HIV infected and uninfected patients with diarrhea in Mozambique. *PLOS Glob Public Health* 2023;3:e0001877.

146. **Iflah M, Kassem E, Rubinstein U, Goren S, Ephros M, *et al.*** Convulsions in children hospitalized for acute gastroenteritis. *Sci Rep* 2021;11:15874.

147. **Ghoshal U, Tejan N, Sisodia J, Verma S, Prasad N, *et al.*** The utility of multiplex polymerase chain reaction for diagnosis of infectious diarrhoea in a tropical country. *Indian J Med Microbiol* 2021;39:323–327.

148. **Chukwu MO, Abia ALK, Ubomba-Jaswa E, Dewar JB, Obi CL**. Mixed Aetiology of Diarrhoea in Infants Attending Clinics in the North-West Province of South Africa: Potential for Sub-Optimal Treatment. *Pathogens* 2020;9:198.

149. **Metreveli M, Bulia S, Shalamberidze I, Tevzadze L, Tsanava S, *et al.*** Campylobacteriosis, Shigellosis and Salmonellosis in Hospitalized Children with Acute Inflammatory Diarrhea in Georgia. *Pathogens* 2022;11:232.

150. **Flipse J, Duim B, Wallinga JA, de Wijkerslooth LRH, Graaf-van Bloois L van der, *et al.*** A Case of Persistent Diarrhea in a Man with the Molecular Detection of Various Campylobacter species and the First Isolation of candidatus Campylobacter infans. *Pathogens* 2020;9:1003.

151. **Borkakoty B, Jakharia A, Sarmah MD, Hazarika R, Baruah PJ, *et al.*** Prevalence of campylobacter enteritis in children under 5 years hospitalised for diarrhoea in two cities of Northeast India. *Indian J Med Microbiol* 2020;38:32–36.

152. **Valledor S, Valledor I, Gil-Rodríguez MC, Seral C, Castillo J**. Comparison of several Real-Time PCR Kits versus a Culture-dependent Algorithm to Identify Enteropathogens in Stool Samples. *Sci Rep* 2020;10:4301.

153. **Noreen Z, Siddiqui F, Javed S, Wren BW, Bokhari H**. Transmission of multidrug-resistant Campylobacter jejuni to children from different sources in Pakistan. *J Glob Antimicrob Resist* 2020;20:219–224.

154. **Tzani M, Mellou K, Kyritsi M, Kolokythopoulou F, Vontas A, *et al.*** 'Evidence for waterborne origin of an extended mixed gastroenteritis outbreak in a town in Northern Greece, 2019'. *Epidemiol Infect* 2020;149:e83.

155. **Valentini D, Vittucci AC, Grandin A, Tozzi AE, Russo C, *et al.*** Coinfection in acute gastroenteritis predicts a more severe clinical course in children. *Eur J Clin Microbiol Infect Dis* 2013;32:909–915.

156. **Berenger BM, Chui L, Ferrato C, Lloyd T, Li V, *et al.*** Performance of four commercial real-time PCR assays for the detection of bacterial enteric pathogens in clinical samples. *Int J Infect Dis* 2022;114:195–201.

157. **Sidafong S, Tanpowpong P, Boonsathorn S, Anurathapan U, Chantarogh S, *et al.*** PCR-based versus conventional stool tests in children with diarrhea who underwent solid organ transplantation or hematopoietic stem cell transplantation. *Medicine (Baltimore)* 2023;102:e35206.

158. **Boyle N, Podczervinski S, Jordan K, Stednick Z, Butler-Wu S, *et al.*** BACTERIAL FOODBORNE INFECTIONS AFTER HEMATOPOIETIC CELL TRANSPLANTATION. *Biol Blood Marrow Transplant* 2014;20:1856–1861.

159. **Schaumburg F, Froböse N, Köck R**. A comparison of two multiplex-PCR assays for the diagnosis of traveller’s diarrhoea. *BMC Infect Dis* 2021;21:181.

160. **Strakova N, Korena K, Gelbicova T, Kulich P, Karpiskova R**. A Rapid Culture Method for the Detection of Campylobacter from Water Environments. *Int J Environ Res Public Health* 2021;18:6098.

161. **Moreno Y, Botella S, Alonso JL, Ferrús MA, Hernández M, *et al.*** Specific Detection of Arcobacter and Campylobacter Strains in Water and Sewage by PCR and Fluorescent In Situ Hybridization. *Appl Environ Microbiol* 2003;69:1181–1186.

162. **Alexandrino M, Grohmann E, Szewzyk U**. Optimization of PCR-based methods for rapid detection of Campylobacter jejuni, Campylobacter coli and Yersinia enterocolitica serovar 0:3 in wastewater samples. *Water Research* 2004;38:1340–1346.

163. **Kim J, Oh E, Banting GS, Braithwaite S, Chui L, *et al.*** An Improved Culture Method for Selective Isolation of Campylobacter jejuni from Wastewater. *Front Microbiol* 2016;7:1345.

164. **Ugarte-Ruiz M, Florez-Cuadrado D, Wassenaar TM, Porrero MC, Domínguez L**. Method Comparison for Enhanced Recovery, Isolation and Qualitative Detection of C. jejuni and C. coli from Wastewater Effluent Samples. *Int J Environ Res Public Health* 2015;12:2749–2764.

165. **Zhang S, Shi J, Li X, Coin L, O’Brien JW, *et al.*** Triplex qPCR assay for Campylobacter jejuni and Campylobacter coli monitoring in wastewater. *Science of The Total Environment* 2023;892:164574.

166. **Zheng T, Li W, Ma Y, Liu J**. Sewers induce changes in the chemical characteristics, bacterial communities, and pathogen distribution of sewage and greywater. *Environmental Research* 2020;187:109628.

167. **Bonetta Si, Pignata C, Lorenzi E, De Ceglia M, Meucci L, *et al.*** Detection of pathogenic Campylobacter, E. coli O157:H7 and Salmonella spp. in wastewater by PCR assay. *Environ Sci Pollut Res* 2016;23:15302–15309.

168. **Strakova N, Shagieva E, Ovesna P, Korena K, Michova H, *et al.*** The effect of environmental conditions on the occurrence of Campylobacter jejuni and Campylobacter coli in wastewater and surface waters. *J Appl Microbiol* 2022;132:725–735.

169. **Soto-Beltrán, M.; Lee, B.G.; Amézquita-López, B.A.; Quiñones, B.** Overview of Methodologies for the Culturing, Recovery and Detection of Campylobacter. *International Journal of Environmental Health Research* **2023**

170. **Joshi MS, Bhalla S, Kalrao VR, Dhongade RK, Chitambar SD**. Exploring the concurrent presence of hepatitis A virus genome in serum, stool, saliva, and urine samples of hepatitis A patients. *Diagnostic Microbiology and Infectious Disease* 2014;78:379–382.

171. **Lee G-Y, Kim W-K, Cho S, Park K, Kim J, *et al.*** Genotyping and Molecular Diagnosis of Hepatitis A Virus in Human Clinical Samples Using Multiplex PCR-Based Next-Generation Sequencing. *Microorganisms* 2022;10:100.

172. **Barathidasan R, Vanathy K, Venkatesh K, Sharmila FM, Dhodapkar R**. Epidemiological and molecular investigation of a hepatitis A outbreak in Tamil Nadu, Southern India. *J Infect Dev Ctries* 2020;14:1475–1479.

173. **Kozak RA, Rutherford C, Richard-Greenblatt M, Chau NYE, Cabrera A, *et al.*** Development and Evaluation of a Molecular Hepatitis A Virus Assay for Serum and Stool Specimens. *Viruses* 2022;14:159.

174. **Poovorawan Y, Theamboonlers A, Chongsrisawat V, Jantaradsamee P, Chutsirimongkol S, *et al.*** Clinical features and molecular characterization of hepatitis A virus outbreak in a child care center in Thailand. *J Clin Virol* 2005;32:24–28.

175. **Lee G-Y, Park K, Lee Y-S, Kim JH, Byun KS, *et al.*** Molecular diagnosis of patients with hepatitis A virus infection using amplicon-based nanopore sequencing. *PLoS One* 2023;18:e0288361.

176. **Bazzardi R, Dore E, Ciccozzi M, Lai A, Pisanu M, *et al.*** Outbreak of acute hepatitis A associated with men who have sex with men (MSM) in North Sardinia 2017-2018. *J Infect Dev Ctries* 2020;14:1065–1070.

177. **Ruchusatsawat K, Wongpiyabovorn J, Kawidam C, Thiemsing L, Sangkitporn S, *et al.*** An Outbreak of Acute Hepatitis Caused by Genotype IB Hepatitis A Viruses Contaminating the Water Supply in Thailand. *Intervirology* 2016;59:197–203.

178. **Mariojoules J, Castro G, Pisano MB, Barbero P, Fantilli A, *et al.*** Hepatitis A outbreak affecting men who have sex with men (MSM) in central Argentina, occurred in July 2017-April 2018, later than the European outbreak. *J Clin Virol* 2019;117:49–53.

179. **Forbi JC, Agwale SM, Ndip LM, Esona MD**. Genetic analysis of hepatitis A virus variants circulating among children presenting with acute diarrhea in Cameroon. *J Med Virol* 2012;84:728–732.

180. **Lo Castro I, Espul C, de Paula VS, Altabert NR, Gonzalez JE, *et al.*** High prevalence of hepatitis A and E viruses in environmental and clinical samples from West Argentina. *Braz J Infect Dis* 2023;27:102738.

181. **Beji-Hamza A, Taffon S, Mhalla S, Lo Presti A, Equestre M, *et al.*** Migration pattern of hepatitis A virus genotype IA in North-Central Tunisia. *Virol J* 2015;12:17.

182. **Hamza H, Abd-Elshafy DN, Fayed SA, Bahgat MM, El-Esnawy NA, *et al.*** Detection and characterization of hepatitis A virus circulating in Egypt. *Arch Virol* 2017;162:1921–1931.

183. **Schlindwein AD, Rigotto C, Simões CMO, Barardi CRM**. Detection of enteric viruses in sewage sludge and treated wastewater effluent. *Water Science and Technology* 2010;61:537–544.

184. **Tsai YL, Tran B, Sangermano LR, Palmer CJ**. Detection of poliovirus, hepatitis A virus, and rotavirus from sewage and ocean water by triplex reverse transcriptase PCR. *Applied and Environmental Microbiology* 1994;60:2400–2407.

185. **Jebri S, Jofre J, Barkallah I, Saidi M, Hmaied F**. Presence and fate of coliphages and enteric viruses in three wastewater treatment plants effluents and activated sludge from Tunisia. *Environ Sci Pollut Res* 2012;19:2195–2201.

186. **Villar LM, De Paula VS, Diniz-Mendes L, Guimarães FR, Ferreira FFM, *et al.*** Molecular detection of hepatitis A virus in urban sewage in Rio de Janeiro, Brazil. *Letters in Applied Microbiology* 2007;45:168–173.

187. **Janahi EM, Mustafa S, Parkar SFD, Naser HA, Eisa ZM**. Detection of Enteric Viruses and Bacterial Indicators in a Sewage Treatment Center and Shallow Water Bay. *Int J Environ Res Public Health* 2020;17:6483.

188. **Hellmér M, Paxéus N, Magnius L, Enache L, Arnholm B, *et al.*** Detection of Pathogenic Viruses in Sewage Provided Early Warnings of Hepatitis A Virus and Norovirus Outbreaks. *Appl Environ Microbiol* 2014;80:6771–6781.

189. **Fantilli A, Cola GD, Castro G, Sicilia P, Cachi AM, *et al.*** Hepatitis A virus monitoring in wastewater: A complementary tool to clinical surveillance. *Water Research* 2023;241:120102.

190. **Béji-Hamza A, Khélifi-Gharbi H, Hassine-Zaafrane M, Della Libera S, Iaconelli M, *et al.*** Qualitative and Quantitative Assessment of Hepatitis A Virus in Wastewaters in Tunisia. *Food Environ Virol* 2014;6:246–252.

191. **Iaconelli M, Purpari G, Della Libera S, Petricca S, Guercio A, *et al.*** Hepatitis A and E Viruses in Wastewaters, in River Waters, and in Bivalve Molluscs in Italy. *Food Environ Virol* 2015;7:316–324.

192. **Prado T, Fumian TM, Miagostovich MP, Gaspar AMC**. Monitoring the hepatitis A virus in urban wastewater from Rio de Janeiro, Brazil. *Transactions of The Royal Society of Tropical Medicine and Hygiene* 2012;106:104–109.

193. **Wang H, Neyvaldt J, Enache L, Sikora P, Mattsson A, *et al.*** Variations among Viruses in Influent Water and Effluent Water at a Wastewater Plant over One Year as Assessed by Quantitative PCR and Metagenomics. *Appl Environ Microbiol* 2020;86:e02073-20.

194. **Jamil K, Abdulrazack N, Fakhraldeen S, Kumar V, Al-Subiai S, *et al.*** Detection of pathogenic viruses in the urban wastewater in Kuwait—implications for monitoring viral disease outbreaks. *Environ Monit Assess* 2023;195:406.

195. **Wong K, Onan BM, Xagoraraki I**. Quantification of Enteric Viruses, Pathogen Indicators, and Salmonella Bacteria in Class B Anaerobically Digested Biosolids by Culture and Molecular Methods. *Appl Environ Microbiol* 2010;76:6441–6448.

196. **PINTÓ RM, ALEGRE D, DOMÍNGUEZ A, EL-SENOUSY WM, SÁNCHEZ G, *et al.*** Hepatitis A virus in urban sewage from two Mediterranean countries. *Epidemiol Infect* 2007;135:270–273.

197. **La Rosa G, Della Libera S, Iaconelli M, Ciccaglione AR, Bruni R, *et al.*** Surveillance of hepatitis A virus in urban sewages and comparison with cases notified in the course of an outbreak, Italy 2013. *BMC Infect Dis* 2014;14:419.

198. **Fernandez-Cassi X, Timoneda N, Martínez-Puchol S, Rusiñol M, Rodriguez-Manzano J, *et al.*** Metagenomics for the study of viruses in urban sewage as a tool for public health surveillance. *Science of The Total Environment* 2018;618:870–880.

199. **Ng TFF, Marine R, Wang C, Simmonds P, Kapusinszky B, *et al.*** High Variety of Known and New RNA and DNA Viruses of Diverse Origins in Untreated Sewage. *J Virol* 2012;86:12161–12175.

200. **Gholizadeh, O.; Akbarzadeh, S.; Ghazanfari Hashemi, M.; Gholami, M.; Amini, P.; Yekanipour, Z.; Tabatabaie, R.; Yasamineh, S.; Hosseini, P.; Poortahmasebi, V.** Hepatitis A: Viral Structure, Classification, Life Cycle, Clinical Symptoms, Diagnosis Error, and Vaccination. *Canadian Journal of Infectious Diseases and Medical Microbiology* **2023**, *2023*, 4263309, doi:10.1155/2023/4263309.

201. **Farzi N, Abrehdari-Tafreshi Z, Zarei O, Chamani-Tabriz L**. Detection of Legionella Pneumophila in Urine and Serum Specimens of Neutropenic Febrile Patients with Haematological Malignancies. *Int J Hematol Oncol Stem Cell Res* 2017;11:49–53.

202. **Vaccaro L, Gomes TS, Izquierdo F, Magnet A, Llorens Berzosa S, *et al.*** Legionella feeleii: Ubiquitous Pathogen in the Environment and Causative Agent of Pneumonia. *Front Microbiol* 2021;12:707187.

203. **Murdoch DR, Walford EJ, Jennings LC, Light GJ, Schousboe MI, *et al.*** Use of the polymerase chain reaction to detect Legionella DNA in urine and serum samples from patients with pneumonia. *Clin Infect Dis* 1996;23:475–480.

204. **Matsiota-Bernard P, Waser S, Vrioni G**. Detection of Legionella pneumophila DNA in urine and serum samples from patients with pneumonia. *Clin Microbiol Infect* 2000;6:223–225.

205. **Murdoch DR, Chambers ST**. Detection of Legionella DNA in peripheral leukocytes, serum, and urine from a patient with pneumonia caused by Legionella dumoffii. *Clin Infect Dis* 2000;30:382–383.

206. **Angrup A, Chaudhry R, Sharma S, Valavane A, Passi K, *et al.*** Application of real-time quantitative polymerase chain reaction assay to detect Legionella pneumophila in patients of community-acquired pneumonia in a tertiary care hospital. *Indian J Med Microbiol* 2016;34:539–543.

207. **Lund V, Fonahn W, Pettersen JE, Caugant DA, Ask E, *et al.*** Detection of Legionella by cultivation and quantitative real-time polymerase chain reaction in biological waste water treatment plants in Norway. *Journal of Water and Health* 2014;12:543–554.

208. **Caicedo C, Beutel S, Scheper T, Rosenwinkel KH, Nogueira R**. Occurrence of Legionella in wastewater treatment plants linked to wastewater characteristics. *Environ Sci Pollut Res* 2016;23:16873–16881.

209. **Viasus, D.; Gaia, V.; Manzur-Barbur, C.; Carratalà, J.** Legionnaires' Disease: Update on Diagnosis and Treatment. *Infect Dis Ther* **2022**, *11*, 973–986, doi:10.1007/s40121-022-00635-7.

210. **Chen J, Xu Y, Yan H, Zhu Y, Wang L, *et al.*** Sensitive and rapid detection of pathogenic bacteria from urine samples using multiplex recombinase polymerase amplification. *Lab Chip* 2018;18:2441–2452.

211. **Vu TVD, Choisy M, Do TTN, Nguyen VMH, Campbell JI, *et al.*** Antimicrobial susceptibility testing results from 13 hospitals in Viet Nam: VINARES 2016-2017. *Antimicrob Resist Infect Control* 2021;10:78.

212. **Dos Reis RO, Cecconi MC, Timm L, Souza MN, Ikuta N, *et al.*** Salmonella isolates from urine cultures: serotypes and antimicrobial resistance in hospital settings. *Braz J Microbiol* 2019;50:445–448.

213. **Choonara FE, Haldorsen BC, Ndhlovu I, Saulosi O, Maida T, *et al.*** Antimicrobial susceptibility profiles of clinically important bacterial pathogens at the Kamuzu Central Hospital in Lilongwe, Malawi. *Malawi Med J* 2022;34:9–16.

214. **Reis ROD, Souza MN, Cecconi MCP, Timm L, Ikuta N, *et al.*** Increasing prevalence and dissemination of invasive nontyphoidal Salmonella serotype Typhimurium with multidrug resistance in hospitalized patients from southern Brazil. *Braz J Infect Dis* 2018;22:424–432.

215. **Moirongo RM, Lorenz E, Ntinginya NE, Dekker D, Fernandes J, *et al.*** Regional Variation of Extended-Spectrum Beta-Lactamase (ESBL)-Producing Enterobacterales, Fluoroquinolone-Resistant Salmonella enterica and Methicillin-Resistant Staphylococcus aureus Among Febrile Patients in Sub-Saharan Africa. *Front Microbiol* 2020;11:567235.

216. **Cuenca-Arias P, Montaño LA, Villarreal JM, Wiesner M**. Molecular and phenotypic characterization of Salmonella Typhimurium monophasic variant (1,4,[5],12:i:-) from Colombian clinical isolates. *Biomedica* 2020;40:722–733.

217. **Kayode A, Okunrounmu P, Olagbende A, Adedokun O, Hassan A-W, *et al.*** High prevalence of multiple drug resistant enteric bacteria: Evidence from a teaching hospital in Southwest Nigeria. *J Infect Public Health* 2020;13:651–656.

218. **Shimelis T, Tadesse BT, W/Gebriel F, Crump JA, Schierhout G, *et al.*** Aetiology of acute febrile illness among children attending a tertiary hospital in southern Ethiopia. *BMC Infect Dis* 2020;20:903.

219. **Zhang J, Huang Y, Xue P, Zhan Z, Huang Z, *et al.*** A duplex droplet digital PCR assay for Salmonella and Shigella and its application in diarrheal and non-diarrheal samples. *Int J Infect Dis* 2022;120:210–216.

220. **Chirambo AC, Nyirenda TS, Jambo N, Msefula C, Kamng'ona A, *et al.*** Performance of molecular methods for the detection of Salmonella in human stool specimens. *Wellcome Open Res* 2020;5:237.

221. **Tosisa W, Mihret A, Ararsa A, Eguale T, Abebe T**. Prevalence and antimicrobial susceptibility of Salmonella and Shigella species isolated from diarrheic children in Ambo town. *BMC Pediatr* 2020;20:91.

222. **Yue M, Liu D, Li X, Jin S, Hu X, *et al.*** Epidemiology, Serotype and Resistance of Salmonella Isolates from a Children's Hospital in Hangzhou, Zhejiang, China, 2006-2021. *Infect Drug Resist* 2022;15:4735–4748.

223. **Rezaei A, Hashemi FB, Heshteli RR, Rahmani M, Halimi S**. Frequency of Salmonella serotypes among children in Iran: antimicrobial susceptibility, biofilm formation, and virulence genes. *BMC Pediatr* 2022;22:557.

224. **Teh CSJ, Lau MY, Chong CW, Ngoi ST, Chua KH, *et al.*** One-step differential detection of Salmonella enterica serovar Typhi, serovar Paratyphi A and other Salmonella spp. by using a quadruplex real-time PCR assay. *J Microbiol Methods* 2021;183:106184.

225. **Farhan Abbas H**. Molecular Detection of Some Virulence Genes in Salmonella Species Isolated from Clinical Samples in Iraq. *Arch Razi Inst* 2022;77:1741–1747.

226. **Indrajith S, Natarajan S, Thangasamy S, Natesan S**. Drug Resistance, Characterization and Phylogenetic Discrepancy of Salmonella enterica Isolates from Distinct Sources. *Curr Microbiol* 2023;80:314.

227. **Bhat A, Rao SS, Bhat S, Vidyalakshmi K, Dhanashree B**. Molecular diagnosis of bacterial and viral diarrhoea using multiplex-PCR assays: An observational prospective study among paediatric patients from India. *Indian J Med Microbiol* 2023;41:64–70.

228. **Balew M, Kibret M**. Prevalence of enteric bacterial pathogens in diarrheic under-five children and their association with the nutritional status in Bahir Dar Zuria District, Northwest Ethiopia. *BMC Nutr* 2023;9:35.

229. **Akinlabi OC, Nwoko E-SQ, Dada RA, Ekpo S, Omotuyi A, *et al.*** Epidemiology and Risk Factors for Diarrheagenic Escherichia coli Carriage among Children in Northern Ibadan, Nigeria. *Am J Trop Med Hyg* 2023;tpmd220618.

230. **Zhang C-M, Xu L-M, Mou X, Xu H, Liu J, *et al.*** Characterization and evolution of antibiotic resistance of Salmonella in municipal wastewater treatment plants. *Journal of Environmental Management* 2019;251:109547.

231. **Diemert S, Yan T**. Municipal Wastewater Surveillance Revealed a High Community Disease Burden of a Rarely Reported and Possibly Subclinical Salmonella enterica Serovar Derby Strain. *Appl Environ Microbiol* 2020;86:e00814-20.

232. **Diemert S, Yan T**. Clinically Unreported Salmonellosis Outbreak Detected via Comparative Genomic Analysis of Municipal Wastewater Salmonella Isolates. *Appl Environ Microbiol* 2019;85:e00139-19.

233. **Yan T, O'Brien P, Shelton JM, Whelen AC, Pagaling E**. Municipal Wastewater as a Microbial Surveillance Platform for Enteric Diseases: A Case Study for Salmonella and Salmonellosis. *Environ Sci Technol* 2018;52:4869–4877.

234. **Leroy-Freitas D, Machado EC, Torres-Franco AF, Dias MF, Leal CD, *et al.*** Exploring the microbiome, antibiotic resistance genes, mobile genetic element, and potential resistant pathogens in municipal wastewater treatment plants in Brazil. *Science of The Total Environment* 2022;842:156773.

235. **Salih H, Karaynir A, Yalcin M, Oryasin E, Holyavkin C, *et al.*** Metagenomic analysis of wastewater phageome from a University Hospital in Turkey. *Arch Microbiol* 2022;204:353.

236. **Kacprzak M, Fijałkowski K, Grobelak A, Rosikoń K, Rorat A**. Escherichia coli and Salmonella spp. Early Diagnosis and Seasonal Monitoring in the Sewage Treatment Process by EMA-qPCR Method. *Pol J Microbiol* 2015;64:143–148.

237. **Fu S, Zhang Y, Wang R, Deng Z, He F, *et al.*** Longitudinal wastewater surveillance of four key pathogens during an unprecedented large-scale COVID-19 outbreak in China facilitated a novel strategy for addressing public health priorities–A proof of concept study. *Water Research* 2023;247:120751.

238. **Zhou N, Ong A, Fagnant-Sperati C, Harrison J, Kossik A, *et al.*** Evaluation of Sampling and Concentration Methods for Salmonella enterica Serovar Typhi Detection from Wastewater. *Am J Trop Med Hyg* 2023;108:482–491.

239. **Uzzell CB, Abraham D, Rigby J, Troman CM, Nair S, *et al.*** Environmental Surveillance for Salmonella Typhi and its Association With Typhoid Fever Incidence in India and Malawi. *The Journal of Infectious Diseases* 2023;jiad427.

240. **Chirambo, A.C.; Nyirenda, T.S.; Jambo, N.; Msefula, C.; Kamng'ona, A.; Molina, S.; Mandala, W.L.; Heyderman, R.S.; Iturizza-Gomara, M.; Henrion, M.Y.R.; *et al.*** Performance of Molecular Methods for the Detection of Salmonella in Human Stool Specimens. *Wellcome Open Res* **2020**, *5*, 237, doi:10.12688/wellcomeopenres.16305.2.

241. **Kramer H, Kuffel G, Thomas-White K, Wolfe AJ, Vellanki K, *et al.*** Diversity of the midstream urine microbiome in adults with chronic kidney disease. *Int Urol Nephrol* 2018;50:1123–1130.

242. **Ou H, Wang Y, Wang Q, Ma Y, Liu C, *et al.*** Rapid detection of multiple pathogens by the combined loop-mediated isothermal amplification technology and microfluidic chip technology. *Ann Palliat Med* 2021;10:11053–11066.

243. **Bischel HN, Özel Duygan BD, Strande L, McArdell CS, Udert KM, *et al.*** Pathogens and pharmaceuticals in source-separated urine in eThekwini, South Africa. *Water Res* 2015;85:57–65.

244. **Raghavan R, Wang S, Dendukuri N, Kar SS, Mahadevan S, *et al.*** Evaluation of LAMP for detection of Shigella from stool samples in children. *Access Microbiol* 2020;2:acmi000169.

245. **Connor S, Velagic M, Zhang X, Johura F-T, Chowdhury G, *et al.*** Evaluation of a simple, rapid and field-adapted diagnostic assay for enterotoxigenic E. coli and Shigella. *PLoS Negl Trop Dis* 2022;16:e0010192.

246. **Pholwat S, Liu J, Taniuchi M, Haque R, Alam MM, *et al.*** Use of Molecular Methods To Detect Shigella and Infer Phenotypic Resistance in a Shigella Treatment Study. *J Clin Microbiol* 2022;60:e0177421.

247. **Liu J, Almeida M, Kabir F, Shakoor S, Qureshi S, *et al.*** Direct Detection of Shigella in Stool Specimens by Use of a Metagenomic Approach. *J Clin Microbiol* 2018;56:e01374-17.

248. **Taneja N, Mewara A, Kumar A, Mishra A, Zaman K, *et al.*** Antimicrobial resistant Shigella in North India since the turn of the 21st century. *Indian J Med Microbiol* 2022;40:113–118.

249. **Kanwar N, Jackson J, Bardsley T, Pavia A, Bourzac KM, *et al.*** Impact of Rapid Molecular Multiplex Gastrointestinal Pathogen Testing in Management of Children during a Shigella Outbreak. *J Clin Microbiol* 2023;61:e0165222.

250. **Olaniran AO, Nzimande SBT, Mkize NG**. Antimicrobial resistance and virulence signatures of Listeria and Aeromonas species recovered from treated wastewater effluent and receiving surface water in Durban, South Africa. *BMC Microbiol* 2015;15:234.

251. **Odjadjare EEO, Obi LC, Okoh AI**. Municipal Wastewater Effluents as a Source of Listerial Pathogens in the Aquatic Milieu of the Eastern Cape Province of South Africa: A Concern of Public Health Importance. *Int J Environ Res Public Health* 2010;7:2376–2394.

252. **Moreno Y, Ballesteros L, García-Hernández J, Santiago P, González A, *et al.*** Specific detection of viable Listeria monocytogenes in Spanish wastewater treatment plants by Fluorescent In Situ Hybridization and PCR. *Water Research* 2011;45:4634–4640.

253. **Shannon KE, Lee D-Y, Trevors JT, Beaudette LA**. Application of real-time quantitative PCR for the detection of selected bacterial pathogens during municipal wastewater treatment. *Science of The Total Environment* 2007;382:121–129.

254. **Halimeh, F.B.; Rafei, R.; Osman, M.; Kassem, I.I.; Diene, S.M.; Dabboussi, F.; Rolain, J.-M.; Hamze, M.** Historical, Current, and Emerging Tools for Identification and Serotyping of Shigella. *Braz J Microbiol* **2021**, *52*, 2043–2055, doi:10.1007/s42770-021-00573-5..

255. **Osman KM, Mustafa AM, Elhariri M, Abdelhamed GS**. Identification of serotypes and virulence markers of Escherichia coli isolated from human stool and urine samples in Egypt. *Indian J Med Microbiol* 2012;30:308–313.

256. **Jenkins C, Lawson AJ, Cheasty T, Willshaw GA**. Assessment of a real-time PCR for the detection and characterization of verocytotoxigenic Escherichia coli. *J Med Microbiol* 2012;61:1082–1085.

257. **Carroll AM, Cobban E, McNamara EB**. Evaluation of molecular and culture methods to determine the optimum testing strategy for verotoxigenic Escherichia coli in faecal specimens. *Diagn Microbiol Infect Dis* 2016;85:1–5.

258. **Xing JZ, Zhu L, Huang B, Chen J, Gabos S**. Microelectronic-sensing assay to detect presence of Verotoxins in human faecal samples. *J Appl Microbiol* 2012;113:429–437.

259. **Rodwell EV, Simpson A, Chan Y-W, Godbole G, McCarthy ND, *et al.*** The epidemiology of Shiga toxin-producing Escherichia coli O26:H11 (clonal complex 29) in England, 2014-2021. *J Infect* 2023;86:552–562.

260. **Bording-Jorgensen M, Parsons B, Szelewicki J, Lloyd C, Chui L**. Molecular Detection of Non-O157 Shiga Toxin-Producing Escherichia coli (STEC) Directly from Stool Using Multiplex qPCR Assays. *Microorganisms* 2022;10:329.

261. **Lucarelli LI, Alconcher LF, Arias V, Galavotti J**. Duration of fecal shedding of Shiga toxin-producing Escherichia coli among children with hemolytic uremic syndrome. *Arch Argent Pediatr* 2021;119:39–43.

262. **Heydari FE, Bonyadian M, Moshtaghi H, Sami M**. Prevalence and antibiotic resistance profile of Shiga toxin-producing Escherichia coli isolated from diarrheal samples. *Iran J Microbiol* 2020;12:289–295.

263. **Singh N, Lapierre P, Quinlan TM, Halse TA, Wirth S, *et al.*** Whole-Genome Single-Nucleotide Polymorphism (SNP) Analysis Applied Directly to Stool for Genotyping Shiga Toxin-Producing Escherichia coli: an Advanced Molecular Detection Method for Foodborne Disease Surveillance and Outbreak Tracking. *J Clin Microbiol* 2019;57:e00307-19.

264. **Omotade TI, Babalola TE, Anyabolu CH, Japhet MO**. Rotavirus and bacterial diarrhoea among children in Ile-Ife, Nigeria: Burden, risk factors and seasonality. *PLoS One* 2023;18:e0291123.

265. **Amin MA, Hashem HR, El-Mahallawy HS, Abdelrahman AA, Zaki HM, *et al.*** Characterization of enterohemorrhagic Escherichia coli from diarrhoeic patients with particular reference to production of Shiga-like toxin. *Microb Pathog* 2022;166:105538.

266. **Eybpoosh S, Mostaan S, Gouya MM, Masoumi-Asl H, Owlia P, *et al.*** Frequency of five Escherichia Coli pathotypes in Iranian adults and children with acute diarrhea. *PLoS One* 2021;16:e0245470.

267. **Loconsole D, Giordano M, Centrone F, Accogli M, Casulli D, *et al.*** Epidemiology of Shiga Toxin-Producing Escherichia coli Infections in Southern Italy after Implementation of Symptom-Based Surveillance of Bloody Diarrhea in the Pediatric Population. *Int J Environ Res Public Health* 2020;17:5137.

268. **Singh P, Metgud SC, Roy S, Purwar S**. Evolution of diarrheagenic Escherichia coli pathotypes in India. *J Lab Physicians* 2019;11:346–351.

269. **Rodwell EV, Chan Y-W, Sawyer C, Carroll A, McNamara E, *et al.*** Shiga toxin-producing Escherichia coli clonal complex 32, including serotype O145:H28, in the UK and Ireland. *J Med Microbiol*;71. Epub ahead of print August 2022. DOI: 10.1099/jmm.0.001579.

270. **Baba H, Kanamori H, Kudo H, Kuroki Y, Higashi S, *et al.*** Genomic analysis of Shiga toxin-producing Escherichia coli from patients and asymptomatic food handlers in Japan. *PLoS One* 2019;14:e0225340.

271. **Kiss C, Kotsanas D, Francis MJ, Sait M, Valcanis M, *et al.*** Molecular epidemiology, clinical features and significance of Shiga toxin detection from routine testing of gastroenteritis specimens. *Pathology* 2023;55:656–662.

272. **Diallo AA, Brugère H, Kérourédan M, Dupouy V, Toutain P-L, *et al.*** Persistence and prevalence of pathogenic and extended-spectrum beta-lactamase-producing Escherichia coli in municipal wastewater treatment plant receiving slaughterhouse wastewater. *Water Research* 2013;47:4719–4729.

273. **Kim, J.; Lee, J.B.; Park, J.; Koo, C.; Lee, M.-S.** Recent Advancements in Technologies to Detect Enterohaemorrhagic Escherichia Coli Shiga Toxins. *J Microbiol Biotechnol* **2023**, *33*, 559–573, doi:10.4014/jmb.2212.12025.

274. **Hayashi H, Uda K, Araki Y, Akahoshi S, Tanaka M, *et al.*** Association of Yersinia Infection With Kawasaki Disease: A Prospective Multicenter Cohort Study. *Pediatr Infect Dis J* 2023;42:1041–1044.

275. **Clarke M, Dabke G, Strakova L, Jenkins C, Saavedra-Campos M, *et al.*** Introduction of PCR testing reveals a previously unrecognized burden of yersiniosis in Hampshire, UK. *J Med Microbiol* 2020;69:419–426.

276. **Kiani P, Bakhshi B, Soltan-Dallal MM, Najar-Peerayeh S**. Heterogeneity of Highly Susceptible Yersinia enterocolitica Isolates of Clinical and Environmental Origin: A 5-Year Survey from Iran (2011-2016). *Microb Drug Resist* 2020;26:46–53.

277. **Rogers WS, Westblade LF, Soave R, Jenkins SG, van Besien K, *et al.*** Impact of a Multiplexed Polymerase Chain Reaction Panel on Identifying Diarrheal Pathogens in Hematopoietic Cell Transplant Recipients. *Clin Infect Dis* 2020;71:1693–1700.

278. **Alnabwani D, Durrani M, Prasad A, Pandya S, Ghodasara K, *et al.*** Yersinia Enterocolitica Sepsis in an Elderly Male With No Iron Overload: A Case Report From the Northeastern United States. *Cureus* 2022;14:e26431.

279. **Rusiñol M, Martínez-Puchol S, Timoneda N, Fernández-Cassi X, Pérez-Cataluña A, *et al.*** Metagenomic analysis of viruses, bacteria and protozoa in irrigation water. *International Journal of Hygiene and Environmental Health* 2020;224:113440.

280. **Shoaib, M.; Shehzad, A.; Raza, H.; Niazi, S.; Mahmood Khan, I.; Akhtar, W.; Safdar, W.; Wang, Z.** A Comprehensive Review on the Prevalence, Pathogenesis and Detection of Yersinia Enterocolitica. *RSC Advances* **2019**, *9*, 41010–41021, doi:10.1039/C9RA06988G..

281. **Ekici A, Gürbüz E, Ünlü AH, Yıldız R, Aydemir S, *et al.*** Investigation of Intestinal and Blood Parasites in People Returning to Turkey with a History of Traveling Abroad During the Pandemic. *Turkiye Parazitol Derg* 2022;46:108–113.

282. **Kinung'hi SM, Magnussen P, Kaatano GM, Kishamawe C, Vennervald BJ**. Malaria and helminth co-infections in school and preschool children: a cross-sectional study in Magu district, north-western Tanzania. *PLoS One* 2014;9:e86510.

283. **Putaporntip C, Buppan P, Jongwutiwes S**. Improved performance with saliva and urine as alternative DNA sources for malaria diagnosis by mitochondrial DNA-based PCR assays. *Clin Microbiol Infect* 2011;17:1484–1491.

284. **Edosomwan EU, Evbuomwan IO, Agbalalah C, Dahunsi SO, Abhulimhen-Iyoha BI**. Malaria coinfection with Neglected Tropical Diseases (NTDs) in children at Internally Displaced Persons (IDP) camp in Benin City, Nigeria. *Heliyon* 2020;6:e04604.

285. **Abuku VG, Allotey EA, Akonde M**. Clinical and laboratory presentation of first-time antenatal care visits of pregnant women in Ghana, a hospital-based study. *PLoS One* 2023;18:e0280031.

286. **Oyeniyi JA, Bello IS, Oyegbade OO, Ibrahim AO, Okunromade OF, *et al.*** Agreement among rapid diagnostic tests, urine malaria tests, and microscopy in

malaria diagnosis of adult patients in southwestern Nigeria. *J Int Med Res* 2022;50:3000605221122740.

287. **Ghayour Najafabadi Z, Oormazdi H, Akhlaghi L, Meamar AR, Raeisi A, *et al.*** Mitochondrial PCR-based malaria detection in saliva and urine of symptomatic patients. *Trans R Soc Trop Med Hyg* 2014;108:358–362.

288. **Imboumy-Limoukou RK, Biteghe-Bi-Essone J-C, Lendongo Wombo JB, Lekana-Douki SE, Rougeron V, *et al.*** Detection of Plasmodium falciparum in Saliva and Stool Samples from Children Living in Franceville, a Highly Endemic Region of Gabon. *Diagnostics (Basel)* 2023;13:3271.

289. **Lompo P, Tahita MC, Sorgho H, Kaboré W, Kazienga A, *et al.*** Pathogens associated with acute diarrhea, and comorbidity with malaria among children under five years old in rural Burkina Faso. *Pan Afr Med J* 2021;38:259.

290. **Keita AK, Fenollar F, Socolovschi C, Ratmanov P, Bassene H, *et al.*** The detection of vector-borne-disease-related DNA in human stool paves the way to large epidemiological studies. *Eur J Epidemiol* 2015;30:1021–1026.

291. **Roth, J.M.; Korevaar, D.A.; Leeflang, M.M.G.; Mens, P.F.** Molecular Malaria Diagnostics: A Systematic Review and Meta-Analysis. *Crit Rev Clin Lab Sci* **2016**, *53*, 87–105, doi:10.3109/10408363.2015.1084991.

292. **Bodur H, Akinci E, Ongürü P, Carhan A, Uyar Y, *et al.*** Detection of Crimean-Congo hemorrhagic fever virus genome in saliva and urine. *Int J Infect Dis* 2010;14:e247-249.

293. **Thomas S, Thomson G, Dowall S, Bruce C, Cook N, *et al.*** Review of Crimean Congo hemorrhagic fever infection in Kosova in 2008 and 2009: prolonged viremias and virus detected in urine by PCR. *Vector Borne Zoonotic Dis* 2012;12:800–804.

294. **Febrer-Sendra B, Fernández-Soto P, García-Bernalt Diego J, Crego-Vicente B, Negredo A, *et al.*** A Novel RT-LAMP for the Detection of Different Genotypes of Crimean-Congo Haemorrhagic Fever Virus in Patients from Spain. *Int J Mol Sci* 2023;24:6411.

295. **Raabe, V.N.** Diagnostic Testing for Crimean-Congo Hemorrhagic Fever. *Journal of Clinical Microbiology* **2020**, *58*, 10.1128/jcm.01580-19, doi:10.1128/jcm.01580-19.

296. **Musso D, Teissier A, Rouault E, Teururai S, de Pina J-J, *et al.*** Detection of chikungunya virus in saliva and urine. *Virol J* 2016;13:102.

297. **Martins EB, Silva MFB, Tassinari WS, de Bruycker-Nogueira F, Moraes ICV, *et al.*** Detection of Chikungunya virus in bodily fluids: The INOVACHIK cohort study. *PLoS Negl Trop Dis* 2022;16:e0010242.

298. **Familiar-Macedo D, Gama BE, Emmel VE, Vera-Lozada G, Abdelhay E, *et al.*** Molecular aspects of Chikungunya virus infections in cancer patients. *Mem Inst Oswaldo Cruz* 2022;117:e210383.

299. **Martins EB, Quintana MSB, Silva MFB, de Bruycker-Nogueira F, Moraes ICV, *et al.*** Predictors of chronic joint pain after Chikungunya virus infection in the INOVACHIK prospective cohort study. *J Clin Virol* 2023;169:105610.

300. **Campos GS, Albuquerque Bandeira AC, Diniz Rocha VF, Dias JP, Carvalho RH, *et al.*** First Detection of Chikungunya Virus in Breast Milk. *Pediatr Infect Dis J* 2017;36:1015–1017.

301. **Kondo M, Akachi S, Ando K, Nomura T, Yamanaka K, *et al.*** Two Japanese siblings affected with Chikungunya fever with different clinical courses: Imported infections from the Cook Islands. *J Dermatol* 2016;43:697–700.

302. **Lee WL, Gu X, Armas F, Leifels M, Wu F, *et al.*** Monitoring human arboviral diseases through wastewater surveillance: Challenges, progress and future opportunities. *Water Research* 2022;223:118904.

303. **Monteiro S, Pimenta R, Nunes F, Cunha MV, Santos R**. Wastewater-based surveillance for tracing the circulation of Dengue and Chikungunya viruses. 2023;2023.10.30.23297765.

304. **Stockdale SR, Blanchard AM, Nayak A, Husain A, Nashine R, *et al.*** RNA-Seq of untreated wastewater to assess COVID-19 and emerging and endemic viruses for public health surveillance. *Lancet Reg Health Southeast Asia* 2023;14:100205.

305. **Johnson, B.W.; Russell, B.J.; Goodman, C.H.** Laboratory Diagnosis of Chikungunya Virus Infections and Commercial Sources for Diagnostic Assays. *The Journal of Infectious Diseases* **2016**, *214*, S471–S474, doi:10.1093/infdis/jiw274.

306. **Andries A-C, Duong V, Ly S, Cappelle J, Kim KS, *et al.*** Value of Routine Dengue Diagnostic Tests in Urine and Saliva Specimens. *PLOS Neglected Tropical Diseases* 2015;9:e0004100.

307. **da Conceição PJP, de Carvalho LR, de Godoy BLV, Nogueira ML, Terzian ACB, *et al.*** Detection of DENV-2 and ZIKV coinfection in southeastern Brazil by serum and urine testing. *Med Microbiol Immunol* 2023;212:193–201.

308. **Pabbaraju K, Wong S, Gill K, Fonseca K, Tipples GA, *et al.*** Simultaneous detection of Zika, Chikungunya and Dengue viruses by a multiplex real-time RT-PCR assay. *Journal of Clinical Virology* 2016;83:66–71.

309. **Choudhury S, Tellier R, Fonseca K, Berenger BM**. Experience with a triplex arbovirus nucleic acid test (NAT) at a Canadian Public Health Laboratory. *BMC Infect Dis* 2021;21:1147.

310. **Humaidi M, Tien WP, Yap G, Chua CR, Ng LC**. Non-Invasive Dengue Diagnostics-The Use of Saliva and Urine for Different Stages of the Illness. *Diagnostics (Basel)* 2021;11:1345.

311. **Barzon L, Gobbi F, Capelli G, Montarsi F, Martini S, *et al.*** Autochthonous dengue outbreak in Italy 2020: clinical, virological and entomological findings. *J Travel Med* 2021;28:taab130.

312. **Van den Bossche D, Cnops L, Van Esbroeck M**. Recovery of dengue virus from urine samples by real-time RT-PCR. *Eur J Clin Microbiol Infect Dis* 2015;34:1361–1367.

313. **Hirayama T, Mizuno Y, Takeshita N, Kotaki A, Tajima S, *et al.*** Detection of dengue virus genome in urine by real-time reverse transcriptase PCR: a laboratory diagnostic method useful after disappearance of the genome in serum. *J Clin Microbiol* 2012;50:2047–2052.

314. **Poloni TR, Oliveira AS, Alfonso HL, Galvão LR, Amarilla AA, *et al.*** Detection of dengue virus in saliva and urine by real time RT-PCR. *Virol J* 2010;7:22.

315. **Ma X, Zhen W, Yang P, Sun X, Nie W, *et al.*** First confirmation of imported dengue virus serotype 2 complete genome in urine from a Chinese traveler returning from India. *Virol J* 2014;11:56.

316. **Sim JXY, Gan ES, Tan HC, Choy MM, Wong HM, *et al.*** Aviremic organ transplant dengue virus transmission - A case report. *Am J Transplant* 2021;21:1944–1947.

317. **Iannetta M, Lalle E, Musso M, Carletti F, Scorzolini L, *et al.*** Persistent detection of dengue virus RNA in vaginal secretion of a woman returning from Sri Lanka to Italy, April 2017. *Euro Surveill* 2017;22:30600.

318. **Zavattoni M, Rovida F, Campanini G, Percivalle E, Sarasini A, *et al.*** Miscarriage following dengue virus 3 infection in the first six weeks of pregnancy of a dengue virus-naive traveller returning from Bali to Italy, April 2016. *Euro Surveill* 2016;21:30308.

319. **Poloni TR, Dornas FP, Dos Santos NN, Soares AM, Amarilla AA, *et al.*** High prevalence of clinically unsuspected dengue disease among children in Ribeirao Preto city, Brazil. *J Med Virol* 2016;88:1711–1719.

320. **Wolfe MK, Paulos AH, Zulli A, Duong D, Shelden B, *et al.*** Wastewater detection of emerging arbovirus infections: Case study of Dengue in the United States. 2023;2023.10.27.23297694.

321. **Raafat, N.; Blacksell, S.D.; Maude, R.J.** A Review of Dengue Diagnostics and Implications for Surveillance and Control. *Transactions of The Royal Society of Tropical Medicine and Hygiene* **2019**, *113*, 653–660, doi:10.1093/trstmh/trz068.

322. **Li M, Wang B, Li L, Wong G, Liu Y, *et al.*** Rift Valley Fever Virus and Yellow Fever Virus in Urine: A Potential Source of Infection. *Virol Sin* 2019;34:342–345.

323. **Haneche F, Leparc-Goffart I, Simon F, Hentzien M, Martinez-Pourcher V, *et al.*** Rift Valley fever in kidney transplant recipient returning from Mali with viral RNA detected in semen up to four months from symptom onset, France, autumn

2015. *Euro Surveill*;21. Epub ahead of print 5 May 2016. DOI: 10.2807/1560-7917.ES.2016.21.18.30222.

324. **Imam IZ, Darwish MA, El-Karamany R**. An epidemic of Rift Valley fever in Egypt. 1. Diagnosis of Rift Valley fever in man. *Bull World Health Organ* 1979;57:437–439.

325. **Lapa, D.; Pauciullo, S.; Ricci, I.; Garbuglia, A.R.; Maggi, F.; Scicluna, M.T.; Tofani, S.** Rift Valley Fever Virus: An Overview of the Current Status of Diagnostics. *Biomedicines* **2024**, *12*, 540, doi:10.3390/biomedicines12030540.

326. **Nagy A, Nagy O, Tarcsai K, Farkas Á, Takács M**. First detection of tick-borne encephalitis virus RNA in clinical specimens of acutely ill patients in Hungary. *Ticks and Tick-borne Diseases* 2018;9:485–489.

327. **Veje M, Studahl M, Norberg P, Roth A, Möbius U, *et al.*** Detection of tick-borne encephalitis virus RNA in urine. *J Clin Microbiol* 2014;52:4111–4112.

328. **Caracciolo I, Bassetti M, Paladini G, Luzzati R, Santon D, *et al.*** Persistent viremia and urine shedding of tick-borne encephalitis virus in an infected immunosuppressed patient from a new epidemic cluster in North-Eastern Italy. *J Clin Virol* 2015;69:48–51.

329. **European Centre for Disease Prevention and Control**. Factsheet about tick-borne encephalitis (TBE). https://www.ecdc.europa.eu/en/tick-borne-encephalitis/facts/factsheet (2017, accessed 25 April 2024).

330. **Barzon L, Pacenti M, Franchin E, Pagni S, Martello T, *et al.*** Excretion of West Nile Virus in Urine During Acute Infection. *The Journal of Infectious Diseases* 2013;208:1086–1092.

331. **Lustig Y, Mannasse B, Koren R, Katz-Likvornik S, Hindiyeh M, *et al.*** Superiority of West Nile Virus RNA Detection in Whole Blood for Diagnosis of Acute Infection. *Journal of Clinical Microbiology* 2016;54:2294–2297.

332. **Tesh RB, Siirin M, Guzman H, Travassos da Rosa APA, Wu X, *et al.*** Persistent West Nile virus infection in the golden hamster: studies on its mechanism and possible implications for other flavivirus infections. *J Infect Dis* 2005;192:287–295.

333. **Gdoura M, Fares W, Bougatef S, Inoubli A, Touzi H, *et al.*** The value of West Nile virus RNA detection by real-time RT-PCR in urine samples from patients with neuroinvasive forms. *Arch Microbiol* 2022;204:238.

334. **Knap N, Korva M, Ivović V, Kalan K, Jelovšek M, *et al.*** West Nile Virus in Slovenia. *Viruses* 2020;12:720.

335. **Nagy A, Bán E, Nagy O, Ferenczi E, Farkas Á, *et al.*** Detection and sequencing of West Nile virus RNA from human urine and serum samples during the 2014 seasonal period. *Arch Virol* 2016;161:1797–1806.

336. **Papa A, Testa T, Papadopoulou E**. Detection of West Nile virus lineage 2 in the urine of acute human infections. *J Med Virol* 2014;86:2142–2145.

337. **Barzon L, Pacenti M, Sinigaglia A, Berto A, Trevisan M, *et al.*** West Nile virus infection in children. *Expert Rev Anti Infect Ther* 2015;13:1373–1386.

338. **Cvjetković IH, Radovanov J, Kovačević G, Turkulov V, Patić A**. Diagnostic value of urine qRT-PCR for the diagnosis of West Nile virus neuroinvasive disease. *Diagn Microbiol Infect Dis* 2023;107:115920.

339. **Gorchakov R, Gulas-Wroblewski BE, Ronca SE, Ruff JC, Nolan MS, *et al.*** Optimizing PCR Detection of West Nile Virus from Body Fluid Specimens to Delineate Natural History in an Infected Human Cohort. *Int J Mol Sci* 2019;20:1934.

340. **Nagy A, Nagy O, Bán E, Molnár E, Müller Z, *et al.*** [Detection of West Nile virus in human samples: follow-up studies during the 2015 seasonal period]. *Orv Hetil* 2017;158:791–796.

341. **Tonry JH, Brown CB, Cropp CB, Co JKG, Bennett SN, *et al.*** West Nile virus detection in urine. *Emerg Infect Dis* 2005;11:1294–1296.

342. **Kuhn KG, Shelton K, Sanchez GJ, Zamor RM, Bohanan K, *et al.*** Wastewater Detection of Emerging Vector-Borne Diseases: West Nile Virus in Oklahoma. Epub ahead of print 2024. DOI: 10.2139/ssrn.4805820.

343. **Barzon, L.; Pacenti, M.; Ulbert, S.; Palù, G.** Latest Developments and Challenges in the Diagnosis of Human West Nile Virus Infection. *Expert Review of Anti-infective Therapy* **2015**, *13*, 327–342, doi:10.1586/14787210.2015.1007044.

344. **Domingo C, Yactayo S, Agbenu E, Demanou M, Schulz AR, *et al.*** Detection of Yellow Fever 17D Genome in Urine. *Journal of Clinical Microbiology* 2020;49:760–762.

345. **Barbosa CM, Paola ND, Cunha MP, Rodrigues-Jesus MJ, Araujo DB, *et al.*** Yellow Fever Virus DNA in Urine and Semen of Convalescent Patient, Brazil - Volume 24, Number 1—January 2018 - Emerging Infectious Diseases journal - CDC. DOI: 10.3201/eid2401.171310.

346. **de Rezende IM, Oliveira GFG, Costa TA, Khan A, Pereira LS, *et al.*** Yellow Fever Molecular Diagnosis Using Urine Specimens during Acute and Convalescent Phases of the Disease. *J Clin Microbiol* 2022;60:e0025422.

347. **Cui S, Pan Y, Lyu Y, Liang Z, Li J, *et al.*** Detection of yellow fever virus genomes from four imported cases in China. *Int J Infect Dis* 2017;60:93–95.

348. **Domingo, C.; Charrel, R.N.; Schmidt-Chanasit, J.; Zeller, H.; Reusken, C.** Yellow Fever in the Diagnostics Laboratory. *Emerging Microbes & Infections* **2018**, *7*, 1–15, doi:10.1038/s41426-018-0128-8.

349. **Lin S-C, Carey BD, Callahan V, Lee J-H, Bracci N, *et al.*** Use of Nanotrap particles for the capture and enrichment of Zika, chikungunya and dengue viruses in urine. *PLoS One* 2020;15:e0227058.

350. **Calvet GA, Kara EO, Bôtto-Menezes CHA, da Costa Castilho M, de Oliveira Franca RF, *et al.*** Detection and persistence of Zika virus in body fluids and associated factors: a prospective cohort study. *Sci Rep* 2023;13:21557.

351. **Lamb LE, Bartolone SN, Chancellor MB**. Detection of Zika Virus Using Reverse Transcription-Loop-Mediated Isothermal Amplification (RT-LAMP). *Methods Mol Biol* 2020;2142:137–146.

352. **Vedovello D, Witkin SS, Silva ACB, Fajardo TCG, Mello AS, *et al.*** Detection of Zika virus in paired urine and amniotic fluid samples from symptomatic and asymptomatic women and their babies during a disease outbreak: association with neurological symptoms in newborns. *J Neurovirol* 2020;26:70–76.

353. **da Conceição PJP, de Carvalho LR, de Godoy BLV, Nogueira ML, Terzian ACB, *et al.*** Detection of Zika virus in urine from randomly tested individuals in Mirassol, Brazil. *Infection* 2022;50:149–156.

354. **Gourinat A-C, O'Connor O, Calvez E, Goarant C, Dupont-Rouzeyrol M**. Detection of Zika Virus in Urine. *Emerg Infect Dis* 2015;21:84–86.

355. **del Pilar Martinez Viedma M, Puri V, Oldfield LM, Shabman RS, Tan GS, *et al.*** Optimization of qRT-PCR assay for Zika virus detection in human serum and urine. *Virus Res* 2019;263:173–178.

356. **Wong JCC, Tay M, Hapuarachchi HC, Lee B, Yeo G, *et al.*** Case report: Zika surveillance complemented with wastewater and mosquito testing. *eBioMedicine* 2024;101:105020.

357. **Alam, Md.A.; Hasan, Mohd.R.; Anzar, N.; Suleman, S.; Narang, J.** Diagnostic Approaches for the Rapid Detection of Zika Virus–A Review. *Process Biochemistry* **2021**, *101*, 156–168, doi:10.1016/j.procbio.2020.11.009.

358. **Pícha D, Moravcová L, Vaňousová D, Hercogová J, Blechová Z**. DNA persistence after treatment of Lyme borreliosis. *Folia Microbiol (Praha)* 2014;59:115–125.

359. **Goodman JL, Jurkovich P, Kramber JM, Johnson RC**. Molecular detection of persistent Borrelia burgdorferi in the urine of patients with active Lyme disease. *Infect Immun* 1991;59:269–278.

360. **Schmidt B, Muellegger RR, Stockenhuber C, Soyer HP, Hoedl S, *et al.*** Detection of Borrelia burgdorferi-specific DNA in urine specimens from patients with erythema migrans before and after antibiotic therapy. *J Clin Microbiol* 1996;34:1359–1363.

361. **Exner MM, Lewinski MA**. Isolation and detection of Borrelia burgdorferi DNA from cerebral spinal fluid, synovial fluid, blood, urine, and ticks using the Roche

MagNA Pure system and real-time PCR. *Diagn Microbiol Infect Dis* 2003;46:235–240.

362. **Pleyer U, Priem S, Bergmann L, Burmester G, Hartmann C, *et al.*** Detection of Borrelia burgdorferi DNA in urine of patients with ocular Lyme borreliosis. *Br J Ophthalmol* 2001;85:552–555.

363. **Schmidt BL, Aberer E, Stockenhuber C, Klade H, Breier F, *et al.*** Detection of Borrelia burgdorferi DNA by polymerase chain reaction in the urine and breast milk of patients with Lyme borreliosis. *Diagn Microbiol Infect Dis* 1995;21:121–128.

364. **Mercier G, Burckel A, Lucotte G**. Detection of Borrelia burgdorferi DNA by polymerase chain reaction in urine specimens of patients with erythema migrans lesions. *Mol Cell Probes* 1997;11:89–94.

365. **Pauluzzi P, Bonin S, Gonzalez Inchaurraga MA, Stanta G, Trevisan G**. Detection of spirochaetal DNA simultaneously in skin biopsies, peripheral blood and urine from patients with erythema migrans. *Acta Derm Venereol* 2004;84:106–110.

366. **Bergmann AR, Schmidt BL, Derler A-M, Aberer E**. Importance of sample preparation for molecular diagnosis of lyme borreliosis from urine. *J Clin Microbiol* 2002;40:4581–4584.

367. **Liebling MR, Nishio MJ, Rodriguez A, Sigal LH, Jin T, *et al.*** The polymerase chain reaction for the detection of Borrelia burgdorferi in human body fluids. *Arthritis Rheum* 1993;36:665–675.

368. **Williams WV, Callegari P, Freundlich B, Keenan G, Eldridge D, *et al.*** Molecular diagnosis of Borrelia burgdorferi infection (Lyme disease). *DNA Cell Biol* 1992;11:207–213.

369. **Schutzer, S.E.; Body, B.A.; Boyle, J.; Branson, B.M.; Dattwyler, R.J.; Fikrig, E.; Gerald, N.J.; Gomes-Solecki, M.; Kintrup, M.; Ledizet, M. *et al.*** Direct Diagnostic Tests for Lyme Disease. *Clinical Infectious Diseases* **2019**, *68*, 1052–1057, doi:10.1093/cid/ciy614

370. **Rahim S, Sharif MM, Amin MR, Rahman MT, Karim MM**. Real Time PCR-based diagnosis of human visceral leishmaniasis using urine samples. *PLOS Glob Public Health* 2022;2:e0000834.

371. **da Costa Lima MS, Hartkopf ACL, de Souza Tsujisaki RA, Oshiro ET, Shapiro JT, *et al.*** Isolation and molecular characterization of Leishmania infantum in urine from patients with visceral leishmaniasis in Brazil. *Acta Trop* 2018;178:248–251.

372. **Mohapatra S, Samantaray JC, Ghosh A**. A Comparative Study of Serum, Urine and Saliva Using rk39 Strip for the Diagnosis of Visceral Leishmaniasis. *J Arthropod Borne Dis* 2016;10:87–91.

373. **Pessoa-E-Silva R, Mendonça Trajano-Silva LA, Lopes da Silva MA, da Cunha Gonçalves-de-Albuquerque S, de Goes TC, *et al.*** Evaluation of urine for Leishmania infantum DNA detection by real-time quantitative PCR. *J Microbiol Methods* 2016;131:34–41.

374. **Silva MAL da, Medeiros Z, Soares CRP, Silva ED da, Miranda-Filho DB, *et al.*** A comparison of four DNA extraction protocols for the analysis of urine from patients with visceral leishmaniasis. *Rev Soc Bras Med Trop* 2014;47:193–197.

375. **De Brito, R.C.F.; Aguiar-Soares, R.D. de O.; Cardoso, J.M. de O.; Coura-Vital, W.; Roatt, B.M.; Reis, A.B.** Recent Advances and New Strategies in Leishmaniasis Diagnosis. *Appl Microbiol Biotechnol* **2020**, *104*, 8105–8116, doi:10.1007/s00253-020-10846-y.

376. **Vieira, A.R.; Salzer, J.S.; Traxler, R.M.; Hendricks, K.A.; Kadzik, M.E.; Marston, C.K.; Kolton, C.B.; Stoddard, R.A.; Hoffmaster, A.R.; Bower, W.A.; *et al.*** Enhancing Surveillance and Diagnostics in Anthrax-Endemic Countries. *Emerg Infect Dis* **2017**, *23*, S147–S153, doi:10.3201/eid2313.170431

377. **Toh E, Williams JA, Qadadri B, Ermel A, Nelson DE**. Development of a SimpleProbe real-Time PCR Assay for rapid detection and identification of the US novel urethrotropic clade of Neisseria meningitidis ST-11 (US_NmUC). *PLoS One* 2020;15:e0228467.

378. B**atista, R.S.; Gomes, A.P.; Dutra Gazineo, J.L.; Balbino Miguel, P.S.; Santana, L.A.; Oliveira, L.; Geller, M**. Meningococcal Disease, a Clinical and Epidemiological Review. *Asian Pacific Journal of Tropical Medicine* **2017**, *10*, 1019–1029, doi:10.1016/j.apjtm.2017.10.004.

379. **Vaidya VM, Malik SVS, Kaur S, Kumar S, Barbuddhe SB**. Comparison of PCR, Immunofluorescence Assay, and Pathogen Isolation for Diagnosis of Q Fever in Humans with Spontaneous Abortions. *J Clin Microbiol* 2008;46:2038–2044.

380. **Mediannikov O, Fenollar F, Socolovschi C, Diatta G, Bassene H, *et al.*** Coxiella burnetii in humans and ticks in rural Senegal. *PLoS Negl Trop Dis* 2010;4:e654.

381. **Schets FM, de Heer L, de Roda Husman AM**. Coxiella burnetii in sewage water at sewage water treatment plants in a Q fever epidemic area. *International Journal of Hygiene and Environmental Health* 2013;216:698–702.

382. **Wielders, C.C.H.; Morroy, G.; Wever, P.C.; Coutinho, R.A.; Schneeberger, P.M.; van der Hoek, W.** Strategies for Early Detection of Chronic Q-Fever: A Systematic Review. *European Journal of Clinical Investigation* **2013**, *43*, 616–639, doi:10.1111/eci.12073.

383. **Hao NV, Huyen NNM, Ny NTH, Trang VTN, Hoang NVM, *et al.*** The Role of the Gastrointestinal Tract in Toxigenic Clostridium tetani Infection: A Case-Control Study. *Am J Trop Med Hyg* 2021;105:494–497.

384. **Basu, S.; Shetty, A.** Laboratory Diagnosis of Tropical Infections. *Indian J Crit Care Med* **2021**, *25*, S122–S126, doi:10.5005/jp-journals-10071-23813.

385. **Kossadoum, R.F.; Baron, A.; Parizot, M.; Husain, M.; Poey, N.; Maurin, M.; Caspar, Y.; Caseris, M.; Bidet, P.; Bonacorsi, S.** Tularemia in Pediatric Patients: A Case Series and Review of the Literature. *The Pediatric Infectious Disease Journal* **2025**, *44*, 180, doi:10.1097/INF.0000000000004554.

386. **Çelebi G, Öztoprak N, Öktem İMA, Heyman P, Lundkvist Å, *et al.*** Dynamics of Puumala hantavirus outbreak in Black Sea Region, Turkey. *Zoonoses Public Health* 2019;66:783–797.

387. **Reynes J-M, Schaeffer L, Papadopoulos P, Ait-Ahmed M, Siby-Diakite D, *et al.*** Molecular Detection of Orthohantavirus puumalaense in Plasma and Urine Samples from Hospitalized Patients Presenting with a Serologically Confirmed Acute Hantavirus Infection in France. *J Clin Microbiol* 2023;61:e0037223.

388. **Steininger P, Herbst L, Bihlmaier K, Willam C, Körper S, *et al.*** Fatal Puumala Hantavirus Infection in a Patient with Common Variable Immunodeficiency (CVID). *Microorganisms* 2023;11:283.

389. **Seo J-W, Kim DY, Kim C-M, Yun N-R, Lee Y-M, *et al.*** Utility of Nested Reverse-Transcriptase Polymerase Chain Reaction of Clinical Specimens for Early Diagnosis of Hemorrhagic Fever with Renal Syndrome. *Am J Trop Med Hyg* 2021;105:1285–1289.

390. M**ir, M.** Hantaviruses. *Clin Lab Med* **2010**, *30*, 67–91, doi:10.1016/j.cll.2010.01.004.
